# Rational Design and Adaptive Management of Combination Therapies for Hepatitis C Virus Infection

**DOI:** 10.1371/journal.pcbi.1004040

**Published:** 2015-06-30

**Authors:** Ruian Ke, Claude Loverdo, Hangfei Qi, Ren Sun, James O. Lloyd-Smith

**Affiliations:** 1 Department of Ecology and Evolutionary Biology, University of California, Los Angeles, Los Angeles, California, United States of America; 2 CNRS/UPMC Univ Paris 06, UMR 8237, Laboratoire Jean Perrin LJP, Paris, France; 3 Department of Molecular and Medical Pharmacology, University of California, Los Angeles, Los Angeles, California, United States of America; 4 The Molecular Biology Institute, University of California, Los Angeles, Los Angeles, California, United States of America; 5 Department of Infectious Diseases, Novartis Institutes for BioMedical Research, Emeryville, California, United States of America; 6 Zhejiang University, Hangzhou, China; 7 Fogarty International Center, National Institutes of Health, Bethesda, Maryland, United States of America; University of Zurich, SWITZERLAND

## Abstract

Recent discoveries of direct acting antivirals against Hepatitis C virus (HCV) have raised hopes of effective treatment via combination therapies. Yet rapid evolution and high diversity of HCV populations, combined with the reality of suboptimal treatment adherence, make drug resistance a clinical and public health concern. We develop a general model incorporating viral dynamics and pharmacokinetics/ pharmacodynamics to assess how suboptimal adherence affects resistance development and clinical outcomes. We derive design principles and adaptive treatment strategies, identifying a high-risk period when missing doses is particularly risky for *de novo* resistance, and quantifying the number of additional doses needed to compensate when doses are missed. Using data from large-scale resistance assays, we demonstrate that the risk of resistance can be reduced substantially by applying these principles to a combination therapy of daclatasvir and asunaprevir. By providing a mechanistic framework to link patient characteristics to the risk of resistance, these findings show the potential of rational treatment design.

## Author Summary

Hepatitis C virus (HCV) affects approximately 170 million people world-wide and chronic infections can lead to cirrhosis and liver cancer. New combination therapies of direct acting antivirals have achieved remarkably high cure rates in clinical trials. However, high mutation rates and high diversity of HCV populations, combined with the reality of suboptimal treatment adherence, make drug resistance a clinical and public health concern. By constructing a mechanistic framework to assess the risk of drug resistance, we provide guidelines for rational design and adaptive management of these promising new therapies. In particular, we identify a high-risk period when missing doses is particularly risky, and quantify the number of extra doses needed to compensate when doses are missed. This framework is a step towards developing a tool for clinicians to design combination therapies and adaptively manage treatment regimens to achieve favorable clinical outcomes.

## Introduction

Hepatitis C virus (HCV) affects approximately 170 million people and chronic infections can lead to cirrhosis and hepatocellular carcinoma [1,2]. Recently, development of direct acting antivirals (DAAs) against HCV infection has revolutionized the field of HCV treatment, because of their high potency, broad applicability and mild side effects [3,4]. Combination therapies of DAAs have achieved remarkably high rates of sustained virological response in clinical trials [5–10]. However, most DAAs have relatively low genetic barriers [11–13], with the exceptions of a few pan-genotypic, yet high-cost DAAs [6]. Because of the high intrinsic mutation rate of HCV [14,15] and the resulting high viral diversity [1,16,17], combined with the reality of suboptimal treatment adherence [18,19], viral resistance is still a clinical and public health concern [13,20]. This is especially true for high-risk groups such as patients with psychiatric disorders or depression [21], and in resource-limited settings where patients have limited access to clinical cares and cannot afford the expensive pan-genotypic DAAs with high genetic barriers [22,23].If treatment is not properly managed, resistance could quickly develop to combination therapies and render these new DAAs useless, as observed for other antimicrobial treatments, squandering the potential health gains from these recent breakthroughs [24–26].

Suboptimal patient adherence to dosing regimens is a crucial risk factor for resistance development in both HIV and HCV treatments [18,19,27,28]. Although high rates of sustained virological response have been achieved in clinical trials, adherence levels may vary substantially among the vast population of infected patients, owing to long treatment periods, complicated regimens associated with DAA combination therapies and limited access to health care [18,19,29–31]. Rational design of combination therapy that achieves viral eradication in patients and maximizes the durability of available DAAs in the presence of suboptimal adherence is a research priority [18,32–34]. In addition, theories that guide individualized regimens based on the genetic composition of a patient’s infection and real-time adjustments for missed doses are needed to avoid resistance. Mathematical models are well suited to address this problem. Previous modeling studies for HIV infections have illuminated potential mechanisms underlying treatment failure and explained puzzling clinical observations [35,36]. However, HCV is a curable disease and its infection, goal of treatment and mechanism of resistance differ from HIV in many respects [37], including no known latent reservoir and a finite treatment period to eradicate the virus. Here, by integrating pharmacokinetics/pharmacodynamics (PK/PD) and viral dynamics into mathematical models, we develop the first general theory to assess the impacts of suboptimal adherence on the outcome of DAA-based therapies for HCV infection. We derive design principles that can be generalized to therapies involving different classes and different numbers of drugs. Using large-scale data from in vitro resistance assays and human clinical trials, we apply this framework to a combination therapy of daclatasvir and asunaprevir [38], and derive evidence-based adaptive treatment strategies for treatment protocols over time according to resistance profiles and adherence patterns.

## Results

**Fig 1:**
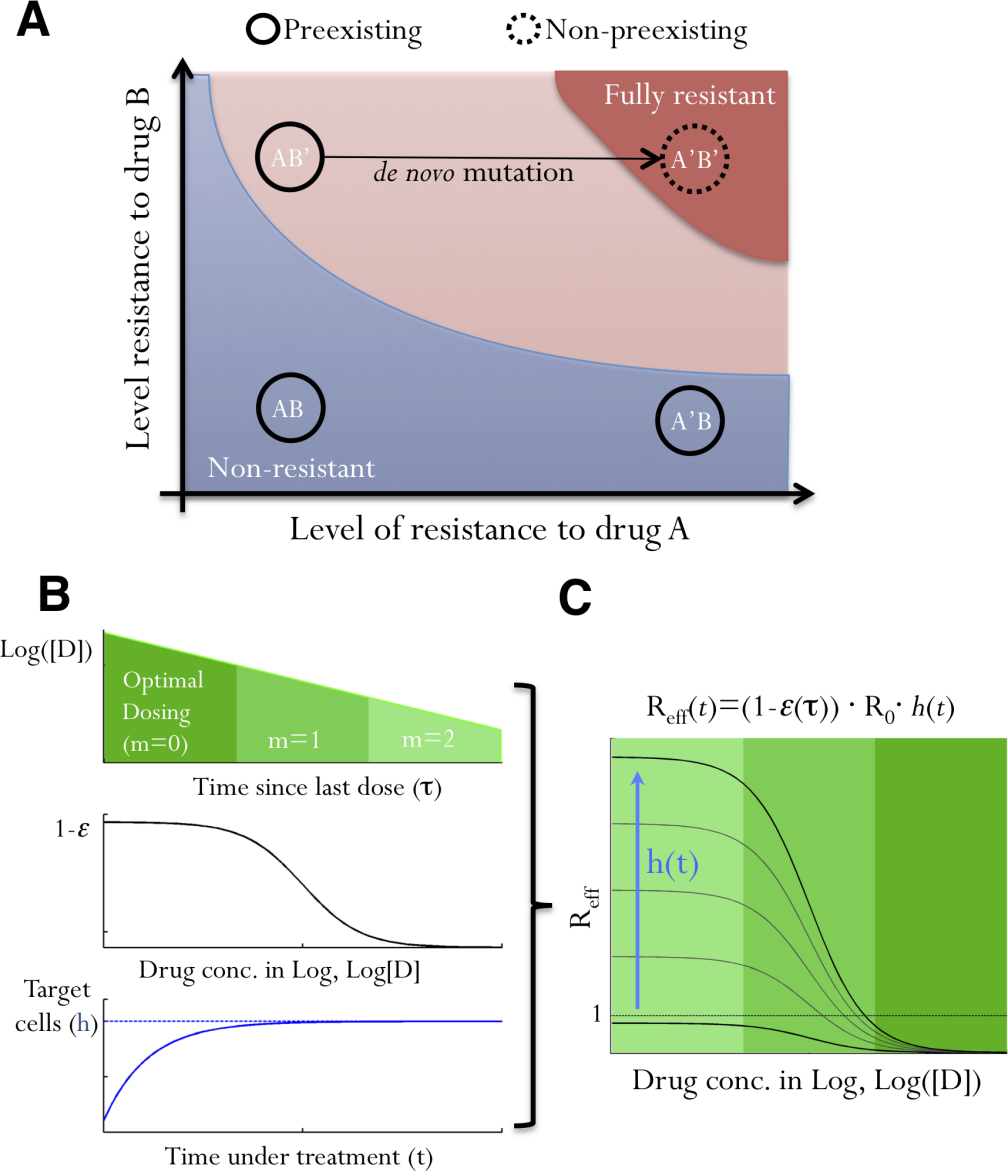
The impacts of suboptimal adherence on viral fitness. **(A)** A schematic illustrating how a non-preexisting mutant, A’B’, fully resistant to a combination therapy involving two drugs, A and B, can be generated when adherence is suboptimal. Each black circle represents a mutant on the parameter space of resistance levels to A and B. AB, A’B and AB’ are preexisting mutants that are non-resistant, resistant to A only and resistant to B only, respectively. Colored areas denote parameter regimes where mutants are fully resistant to the therapy (red), can grow if doses are missed (pink), and do not grow (blue). Note that the pink area can grow or shrink on the parameter space depending on the number of consecutively missed doses and drug PK/PD, and mutants lying in the pink area are ‘partially resistant mutants’. **(B)** The dynamics of viral strains under treatment are determined by several factors: drug concentration, [D], which decreases with an increasing number of missed doses, *m* (upper panel); how viral replication is affected by drug (1-ε; middle panel); and the relative number of target cells, *h*(t) (lower panel). Upon effective treatment, *h*(*t*) increases to the infection-free level. **(C)** We integrate all these factors into a single fitness parameter, *R*_*eff*_(*t*). Viral fitness increases as drug concentration drops (indicated by shades of green) and as target cell abundance rises (the blue arrow). Values of *R*_*eff*_(*t*) can exceed 1, i.e. positive growth, if doses are missed after a period of effective treatment.

Resistance to antiviral treatments can develop through selection of preexisting mutants or *de novo* generation of new mutants. A core principle for designing effective combination therapy is that, if patients fully adhere to the treatment regimen, the treatment must suppress all preexisting mutants and *de novo* resistance should be unlikely [39]. Missing doses, however, can lead to suboptimal drug concentrations, allowing growth of some preexisting mutants with partially resistant phenotypes. Growth of these mutants allows the viral population to survive longer, possibly generating further mutations that contribute *de novo* resistance against the full combination therapy. For example, consider a combination therapy of two DAAs, A and B, as shown in Fig 1A. If missed doses and pharmacokinetics lead to a drop in the concentration of drug A, this allows growth of the preexisting mutant, AB’, (which is already resistant to drug B), thus opening opportunities to generate the fully resistant mutant, A’B’. Therefore, the dynamics of the subset of preexisting mutants that have a high level of resistance against single DAAs determine resistance evolution and treatment outcomes for combination therapies. In the following, we denote these mutants as *‘partially resistant’ mutants*.

### The effective viral fitness, R_eff_(t)

The fitness of a particular strain in a treated patient is determined by the PK/PD of the drug, the level of resistance of the strain, and the availability of target cells, i.e. uninfected hepatocytes for HCV (Fig 1B). We can integrate all these factors (for any class of DAA therapy) into a single number, the effective reproductive number under treatment, *R*_*eff*_(*t*) (Fig 1C). *R*_*eff*_(*t*) is the average number of cells infected by viruses produced by a single infected cell. It acts as a measure of viral fitness, and can be calculated as:

$$R_{eff}\left(t\right)=\left(1-\varepsilon \left(t\right)\right)\cdot R_{0}\cdot h\left(t\right)$$ (1)

where *t* is time since treatment starts, *τ* is the time since last dose, *ε*(*τ*) is the efficacy of the drug combination at time *τ* during the dosing cycle, *R*_0_ is the reproductive number of the virus in the absence of treatment, and *h*(*t*) is the normalized abundance of target cells (see S1 Text). Under effective treatment, the availability of target cells, *h*(*t*), increases quickly to reach the infection-free level [40], and therefore, the overall viral fitness increases over time as *h*(*t*) increases under effective treatment (Fig 1B and 1C). When adherence is optimal (i.e. no missing doses), the value of *R*_*eff*_ for a partially resistant mutant is always less than 1 (i.e. viral suppression); however, if doses are missed, drug concentration declines exponentially and *R*_*eff*_ can become greater than 1 (i.e. viral growth) (Fig 1C). Note that, although we consider the fitness of a single ‘partially resistant’ mutant here, the competition between different mutants is described implicitly in Eq 2 by the target cell availability, *h*(*t*): if a ‘partially resistant’ mutant rises to a high abundance due to missing doses, then *h*(*t*) will decrease to a low level again, leading to decreases in fitness for all viral mutants.

### The growth of partially resistant mutants and the need for extended treatment

**Fig 2:**
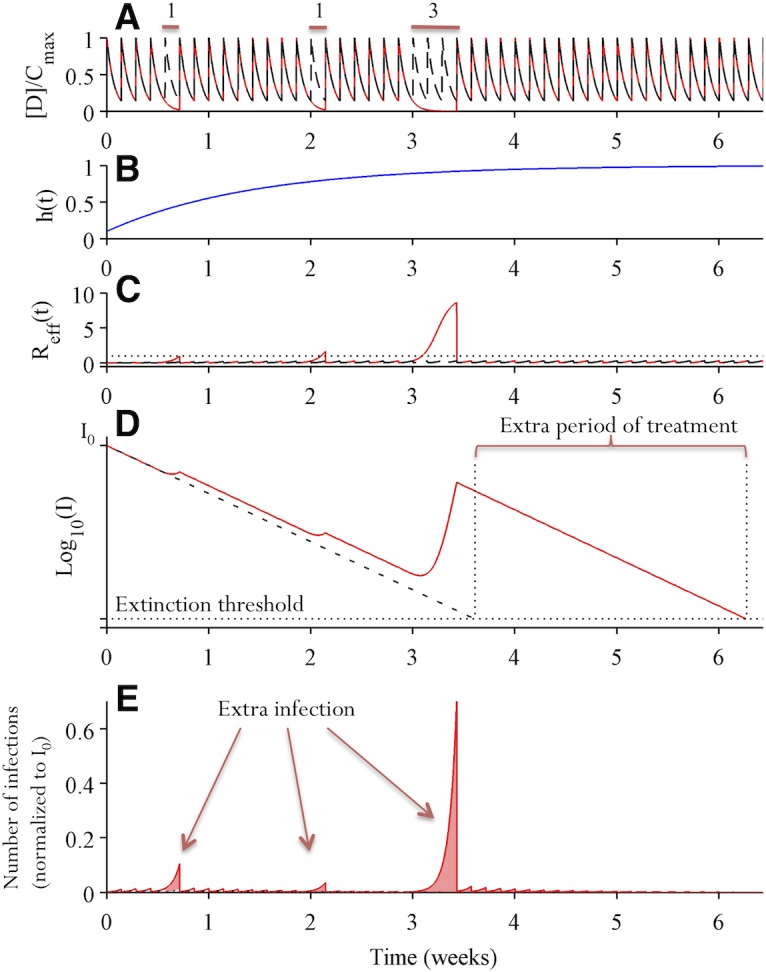
Suboptimal adherence prolongs treatment time needed to eliminate *partially resistant mutants* and increases the risk of *de novo* evolution of fully resistant strains. Two simulations assuming perfect adherence (dashed black lines) and imperfect adherence (solid red lines) are shown. In the simulation assuming imperfect adherence, single doses are missed at day 5 and 15, and 3 consecutive doses are missed during days 22–24. **(A)** Drug concentration over time normalized by the maximum drug concentration *C*_*max*_. **(B)** The abundance of target hepatocytes, which rebounds after the initiation of combination therapy. **(C)** Viral fitness of the partially resistant mutant under consideration. Missing doses increases the value of *R*_*eff*_, especially when multiple consecutive doses are missed or when *h*(*t*) has increased to high levels. **(D)** The dynamics of cells infected by the partially resistant mutant (on log_*10*_ scale). The number of infected cells declines almost exponentially when doses are taken. Missed doses allow the number of infected cells to rebound. This means that an additional period of treatment is needed to suppress the mutant below the extinction threshold level, i.e. to achieve viral elimination. **(E)** The number of cells newly infected by the partially resistant mutants. Missed doses lead to substantial numbers of additional new infections, especially when 3 consecutive doses are missed.

We now consider how suboptimal adherence impacts the dynamics of partially resistant mutants. As an illustration, we contrast simulations assuming perfect adherence versus suboptimal adherence. Missing doses leads to rapid decreases in drug concentration, and thus increases in R_*eff*_ of a partially resistant mutant (Fig 2A–2C). This means that extra doses are needed to compensate for the missed doses to suppress the mutant to extinction (Fig 2D), and also that the number of newly infected cells rises substantially, which increases the opportunity for *de novo* resistance (Fig 2E).

We approximate the time-varying values of *R*_*eff*_(*t*) during periods when doses are missed, by calculating the average effective reproductive number, *R*_*ave*,*m*_, as (see Materials and Methods):

$$R_{ave,m}\left(t\right)\approx \left(1-\varepsilon_{ave,m}\right)\cdot R_{0}\cdot h\left(t\right)$$ (2)

where *t* is the time when the patient starts to miss doses, *m* is the number of consecutive doses missed and *ε*_*ave*,*m*_ is the average drug inhibition during the period when *m* consecutive doses are missed. This allows us to generalize our theory to any DAA combinations for which *ε*_*ave*,*m*_ can be either estimated from pharmacokinetics/pharmacodynamics data or calculated from mutant resistance profiles [36].

We then ask, if *m* consecutive doses are missed beginning at time *t*, how many extra doses, *N*_*m*_, are needed to compensate? This number, which we denote ‘compensatory doses’, can be approximated as (see Materials and Methods):

$$N_{m}\left(t\right)\approx m\cdot R_{ave,m}\left(t\right)\approx m\cdot \left(1-\varepsilon_{ave,m}\right)\cdot R_{0}\cdot h\left(t\right)$$ (3)

This allows us to estimate the total duration of treatment needed to clear infection for a given adherence pattern. Furthermore, since *h*(*t*) increases over time under effective treatment [40], Eq 3 shows that a higher number of extra doses are needed to eliminate the infection if doses are missed later in treatment.

### *De novo* generation of fully resistant mutants

To assess the risk that a partially resistant lineage will give rise to full resistance, we calculate the expected number of target cells, Φ_*m*_, that become infected by fully resistant mutant viruses due to *de novo* mutation during a period when *m* consecutive doses are missed. This quantity is the product of the cumulative number of cells newly infected by a partially resistant mutant and the effective mutation rate from that mutant to the fully resistant mutant, *μ*_*eff*_ (see Materials and Methods):

$$\Phi_{m}\left(t\right)\approx \mu_{eff}•\underset{\text{cumulative number of cells newly infected by a partially resistant mutant}}{\underbrace{I\left(t\right)•\overset{\Theta (t)}{\overbrace{\frac{R_{ave,m}\left(t\right)}{R_{ave,m}\left(t\right)-1}•\left(e^{(R_{ave,m}\left(t\right)-1)•\delta •m•T}-1\right)}}}}$$ (4)

where *I*(*t*) is the number of cells infected by the partially resistant mutant at time *t* when the first dose is missed, and Θ(*t*) represents the potential to generate new infections. *δ* is the death rate of infected hepatocytes, and *T* is the scheduled interval between two doses. Φ_*m*_ quantifies the risk that a fully resistant mutant infects target cells, but whether it emerges and becomes established within the host depends on its fitness and the stochastic dynamics of invasion [41–43].

**Fig 3:**
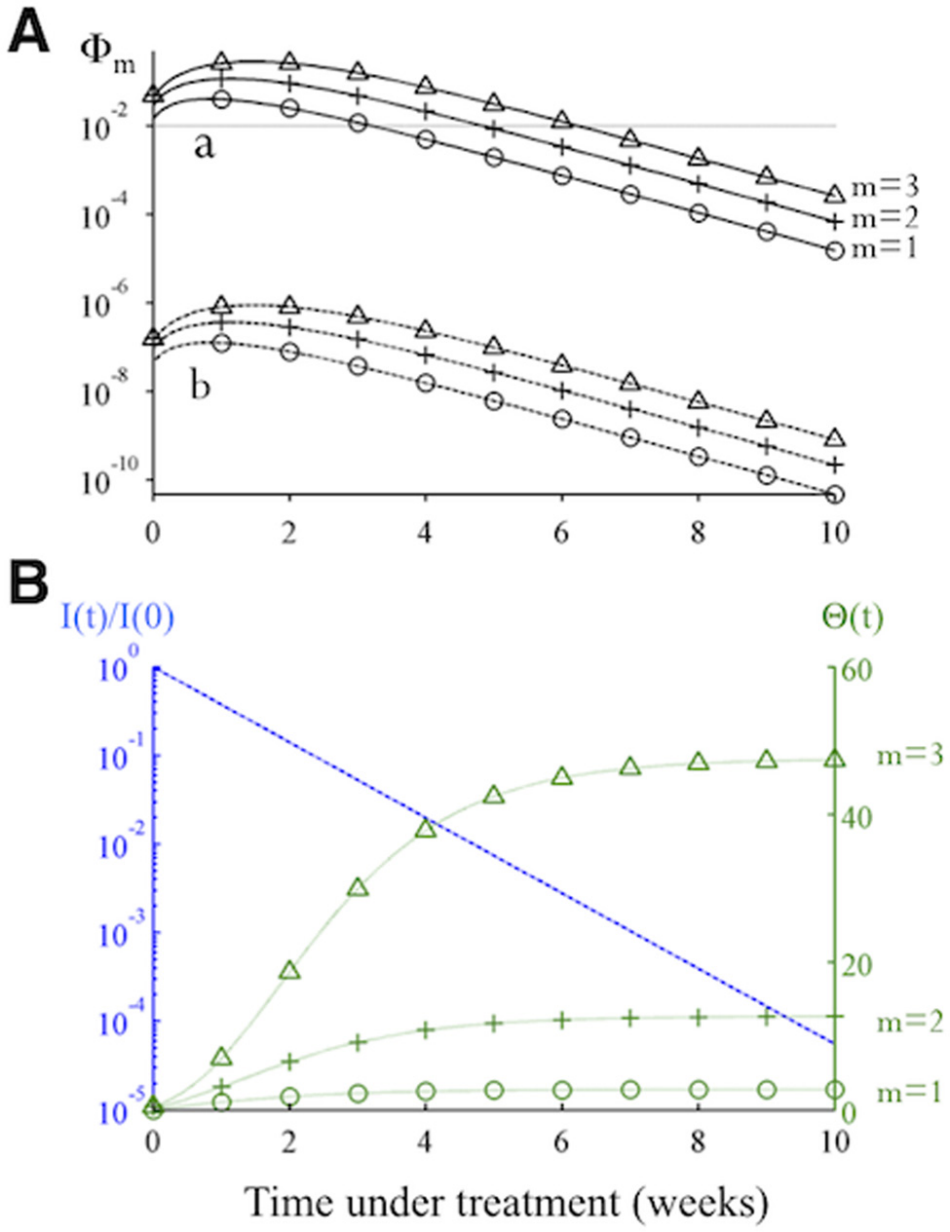
There is a high-risk window early in treatment when missing doses is more likely to cause *de novo* resistance. **(A)** The changes in the risk of *de novo* resistance, Φ_*m*_, generated by a partially resistant mutant over time. The two sets of trajectories, A and B, differ in that the value of *μ*_*eff*_ for trajectory B is smaller by a factor of 10^–5^ (representing one additional nucleotide mutation) than the value set for trajectory A. Each set of trajectories shows the risk when the number of doses missed (*m*) is 1,2 or 3. **(B)** Dynamics of the two time-varying quantities in Eq 4, i.e. the number of cells infected by the partially resistant mutant relative to the initial number before treatment (I(t)/I(0); blue dashed line), and the value of Θ(*t*), green dotted lines, as shown in Eq 4. Under effective treatment, the number of infected cells *I*(*t*) decreases exponentially, while the number of target cells rebounds to the infection-free level quickly, causing an increase in R_*ave*,*m*_ and thus Θ(*t*). Together these changes cause Φ_*m*_ to increase initially and then to decrease exponentially at longer times (as seen in panel A).

The strong dependence of Φ_*m*_ on *μ*_*eff*_ predicts that designing combination therapies to increase the genetic barrier to full resistance, e.g. using DAAs with higher genetic barrier or adding an extra drug into the combination, can reduce Φ_*m*_ by orders of magnitude or more, thus it would lead to drastic reductions in the probability of generating full resistance (compare trajectories a and b in Fig 3A).

Eq 4 also allows us to assess when during treatment it is most risky to miss doses, which can inform treatment guidelines. Changes in two quantities, *I*(*t*) and Θ(*t*), determine changes in Φ_*m*_ over the course of a treatment regimen. For as long as adherence is perfect, *I*(*t*) decreases exponentially, while Θ(*t*) increases over time since *R*_*ave*,*m*_(*t*) increases as the abundance of target cells rises over time (Fig 3B). Thus the value of Φ_*m*_ first increases (due to rapid recovery of target cells) and then decreases exponentially (due to decrease of infected cells). This leads to a high-risk window period, during which missing doses is especially risky for generating full resistance (Fig 3A). This qualitative finding is robust to changes in model parameters, though quantitative predictions of the risk of full resistance depend on the fitness of the mutant (*R*_0_), the half-life of infected cells (*δ*), and the rate at which the target hepatocytes become available upon treatment (S1 Fig).

### Design principles and adaptive treatment strategy for DAA combination therapy

These results suggest principles for designing combination therapies and rational optimization of treatment outcomes. First, the genetic barrier to full resistance to a therapy is an important determinant of the risk of resistance. Assessment of treatment readiness has been a low-cost routine clinical practice for HIV treatment [44]. Similar strategies can be implemented for HCV treatment. Based on the assessment, for patients who are predicted to maintain high adherence, combinations of DAA that ensure the fully resistant mutants are not pre-existing would be sufficient. For patients with risk factors for low adherence, therapies should be designed by selecting drug combinations that impose a higher genetic barrier than required to suppress all pre-existing mutants. Second, we have shown there exists a high-risk window period where the risk of *de novo* resistance is high. Intervention efforts to ensure a high level of adherence during the high-risk window period (indicated by the value of Φ_*m*_) would reduce the risk of resistance and treatment failure. Third, because of the exponential growth of ‘partially resistant mutants’ when doses are missed, missing a number of doses consecutively leads to a much higher risk of *de novo* resistance than missing the same number of doses separately [36]. Thus, missing a block of doses should be avoided.

*Adaptive treatment strategies* could be developed based on the theoretical findings shown above. If doses are missed during treatment, the patient should be treated with extra doses, computed as the maximum value of the *N*_*m*_ values calculated for all partially resistant mutants. For the lowest risk of *de novo* resistance, the prescribed number of compensatory doses (*N*_*m*_) should be taken, uninterrupted, immediately after doses are missed. Otherwise the infected cell population may rebound to a high level, which can make further missed doses very risky for resistance.

### Case study: Combination therapy of daclatasvir and asunaprevir

To demonstrate the practical applicability of our theory, we consider a recently developed interferon-free combination therapy based on an NS5A inhibitor, daclatasvir, and an NS3 protease inhibitor, asunaprevir [38]. In clinical trials, a large proportion of patients infected with HCV genotype-1b achieved sustained virological response (i.e. viral eradication) when treated with daclatasvir and asunaprevir for 24 weeks, although viral breakthrough and viral relapse occurred in a small fraction of patients [45,46].

**Fig 4:**
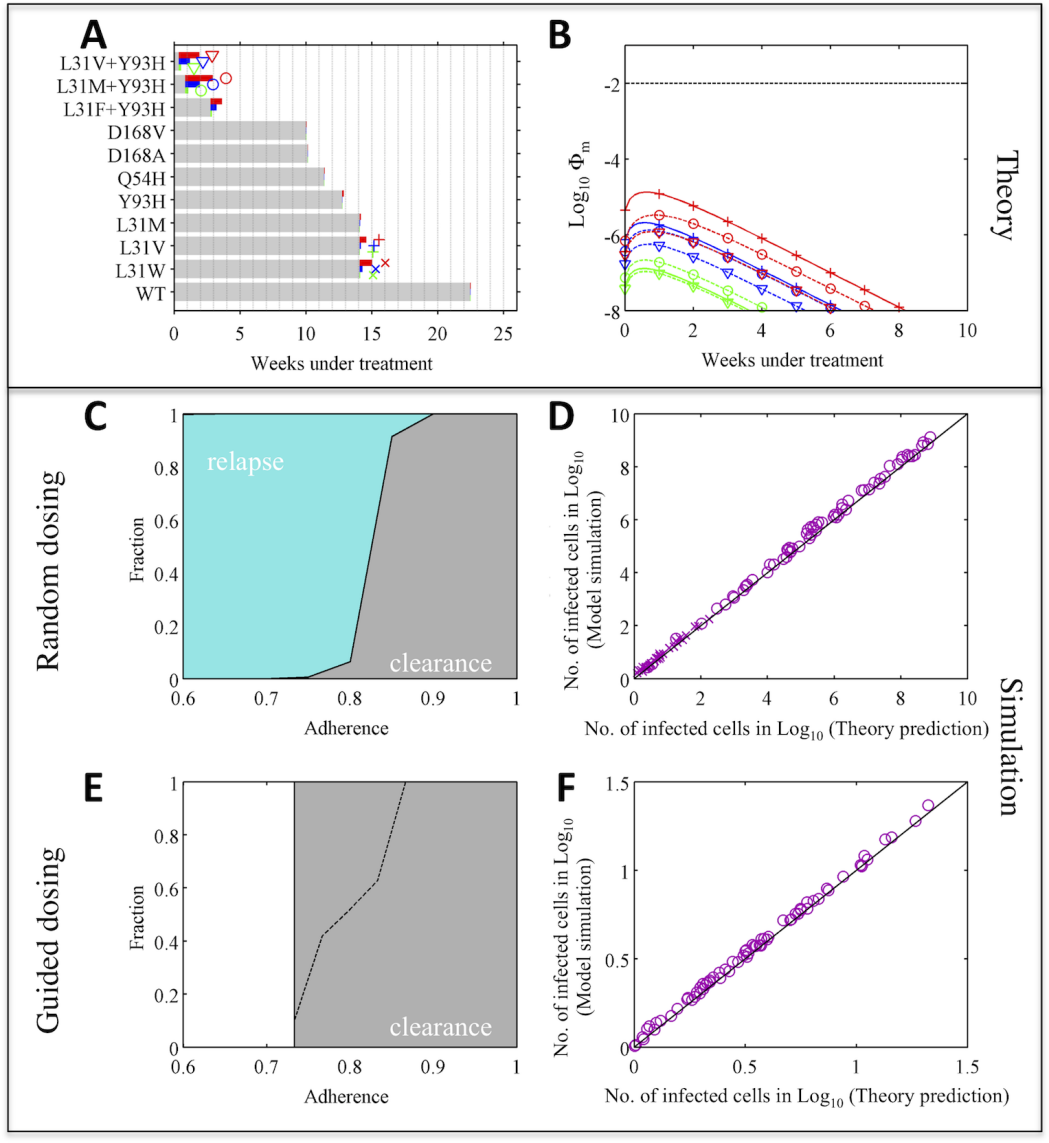
Adaptive treatment strategy improves treatment outcome substantially—a case when the risk of *de novo* resistance is low (with wild-type genotype-1b HCV at baseline). **(A)** Theoretical prediction of the treatment duration needed to eliminate each partially resistant mutant under perfect adherence (gray bar), and the maximum number of additional doses needed to compensate for missing doses, *N*_*m*,*max*_ (colored bars). Green, blue and red denote results when 1, 2 and 3 consecutive daily doses are missed, respectively. The longer the colored bars, the greater the impact of missing doses. The symbols next to the bars for *N*_*m*,*max*_ show the type of mutant investigated in panels (B,D,F). **(B)** Theoretical prediction of the risk of *de novo* resistance, Φ_*m*_, over time (as shown in Fig 3D), for the three mutants with highest risks of generating fully resistant mutants. The dashed black line shows Φ_*m*_ = 0.01. **(C)** Treatment outcomes 1–3 days of doses are missed randomly. Colored areas denote the fractions of simulations with outcomes of viral relapse without full resistance (light blue) and viral clearance (gray). **(D)** Comparison between theory predictions and simulations of the number of cells infected by different mutants after 24 weeks of treatment. **(E)** Treatment outcomes if adaptive treatment strategy is followed. The area above the black dashed line denotes the fraction of patients where virus is not cleared after 24 weeks’ treatment. After 24 weeks, patients take the prescribed number of make-up doses without missing further doses. White areas denote adherence levels that are not allowed by the adaptive treatment strategy. **(F)** Same comparison as in panel (D) for the guided dosing simulation.

We first consider patients with the wild-type virus at baseline, i.e. the wild-type virus is the dominant strain before treatment. Using the PK/PD data for each drug [47–49] and the resistance profiles data measured for genotype-1b HCV [50,51], we predicted which mutants are potentially fully-resistant to this combination therapy and calculated the values of *N*_*m*_ and Φ_*m*_ for each of the partially resistant mutants (Fig 4A and 4B) (see Supplementary Materials for more detail). Choosing the highest values of *N*_*m*_ and Φ_*m*_ among all the partially resistant mutants allows us to project the overall risk arising from missed doses over the course of treatment, and we found required numbers of compensatory doses were modest and the risk of *de novo* resistance is low (S2A Fig). To demonstrate that the theoretical approximations represent the full viral dynamics accurately, we simulated a multi-strain viral dynamics model (see Materials and Methods), assuming 1–3 day blocks of consecutive doses are missed randomly within a treatment regimen lasting 24 weeks. The model predicts that relapse of L31M+Y93H or L31W would be observed when overall adherence is less than 90% (Fig 4C and 4D). Indeed, the L31M+Y93H mutant has already been detected in one relapse patient in a clinical trial [46]. There is excellent agreement between simulation results and theoretical predictions (based on Eqs 3 and 4) for the number of cells infected by different mutants after 24 weeks of treatment and the cumulative number of cells infected by partially resistant mutants over the treatment period (Figs 4D and S3).

We then simulated outcomes when the doses are guided by the adaptive treatment strategy (*guided dosing*; see Methods for detailed simulation procedure). Because the risk of *de novo* resistance when doses are missed is low, there is no high-risk period for *de novo* resistance in this case (Fig 4B). If patient dosing is guided, i.e. all the required doses and the extra doses to compensate for the missed doses are taken, the infection can be cleared successfully (Fig 4E). Again, we find excellent agreement between simulation results and theoretical predictions (Fig 4F).

**Fig 5:**
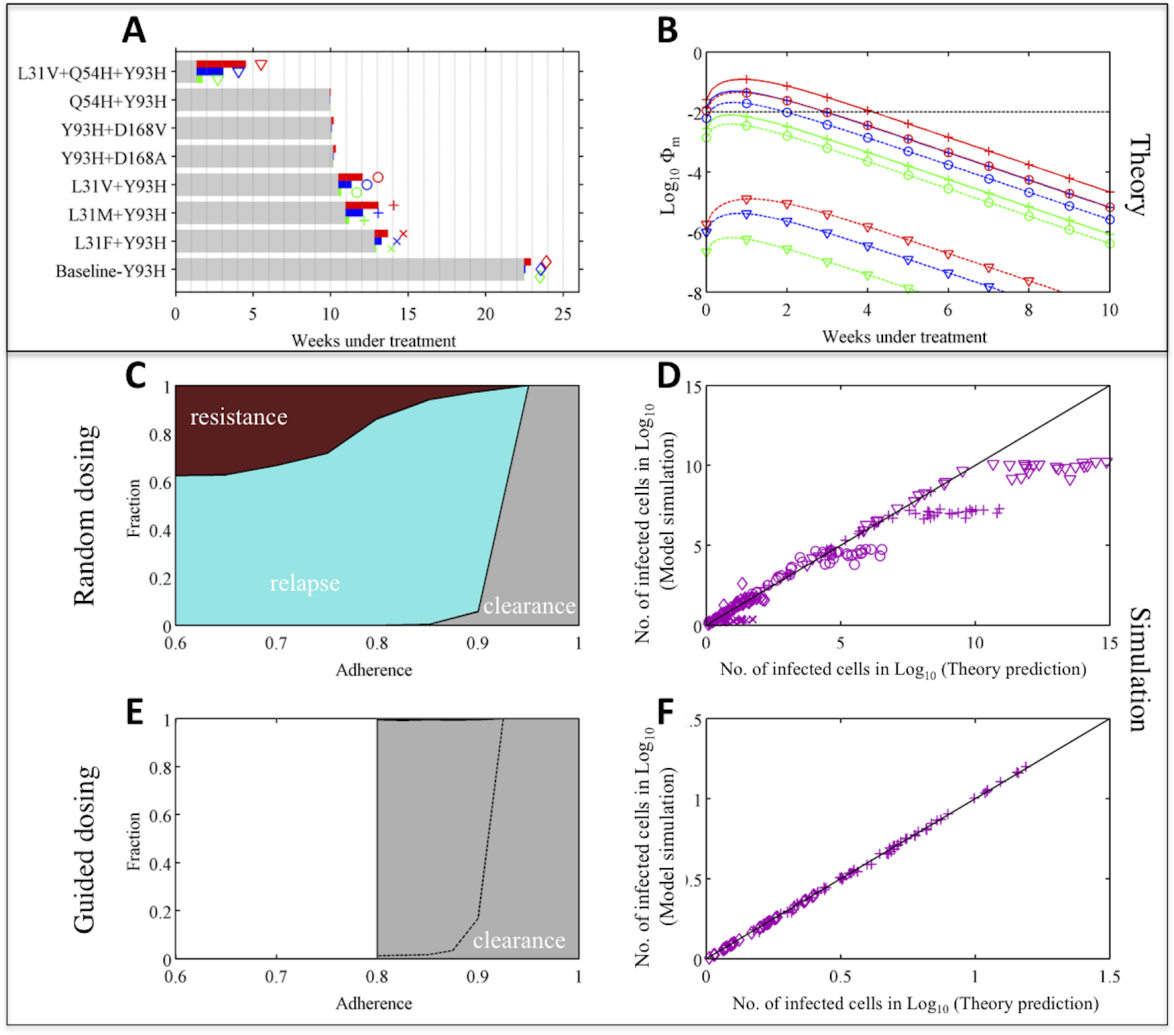
Adaptive treatment strategy prevents *de novo* resistance and improves treatment outcome substantially—a case when the risk of *de novo* resistance is high. Theoretical prediction and simulation for patients with the Y93H mutant virus (genotype-1b) at baseline under combination therapy of daclatasvir and asunaprevir. Thus, the mutants considered here all have the Y93H mutation. The theoretical predictions and simulation results are plotted in the same way as in Fig 4. Dark red areas in panel (C,E) denote the fraction of patients with *de novo* full resistance to the combination therapy. Note that the fraction of patients with *de novo* resistance in the guided dosing scenario is very small (<0.1%). When doses are guided, so that mutant viral load does not rebound to the pre-treatment level, the theoretical prediction agrees well with simulation as shown in panel (F).

Many patients bear the Y93H mutation at baseline and this mutation reduces the genetic barrier to full resistance by one nucleotide[46]. Our theory suggests that reducing the genetic barrier to full resistance will drastically increase the risk of treatment failure. We repeated our analysis for patients with Y93H at baseline, to test how our adaptive treatment strategy works when the risk of resistance is high. As predicted, many more days of treatment are needed to compensate for missed doses, and the risks of generating full resistance *de novo* are high (>0.01) during the first 3 weeks of effective treatment if 2 consecutive doses are missed (or first 4 weeks if 3 doses are missed; Fig 5A and 5B and S2B Fig). *De novo* full resistance is likely if doses are missed randomly and adherence is less than 90% (dark red area in Fig 5C). The predicted number of infected cells agrees well with simulation, except when adherence is very low such that viral load rebounds back close to the pre-treatment level (Fig 5D and S4–S6 Figs). In stark contrast, when doses are guided, the risk of *de novo* resistance becomes much lower (compare Fig 5C with 5E). Again, for patients who do not clear infection after 24-week treatment, extended periods of treatment as predicted by our theory (using Eq 3) can clear infection with low risk of resistance. The efficacy of the adaptive treatment strategy is robust across different parameter values (S7–S12 Figs and S1 Text). Therefore, our treatment strategy can improve clinical outcomes substantially by adjusting on-going treatment based on patient adherence patterns.

## Discussion

In this study, we integrate PK/PD parameters and viral dynamics into a unified framework to assess the impacts of suboptimal treatment adherence on the risk of treatment failure. Using simulations incorporating PK/PD and resistance profile data collected previously [48,50,51], we showed that treatment outcomes of combinations therapies of daclatasvir and asunaprevir can be improved by this adaptive treatment strategy, especially when the Y93H mutant is the dominant strain before treatment begins.

We have identified several factors that influence the risk of *de novo* resistance to a combination therapy. Among these factors, the genetic barrier to full resistance plays a dominant role. Thus, for patients with risk of low adherence, combinations that impose a higher genetic barrier are recommended. This is especially important in resource-limited settings where patients have limited access to health care and adherence is not closely monitored. The recently developed HCV entry inhibitors [52], which inhibit host factors that are required for viral entry (instead of viral factors), may offer a promising direction for HCV combination therapy, because of their high genetic barriers to resistance, and their synergistic interactions with other classes of DAAs. For situations where therapies with low genetic barriers to resistance are used, we have identified a high-risk window period during which *de novo* resistance is likely if doses are missed. Intervention efforts should focus on enhancing patients’ adherence during this period. Additional complementary strategies could further reduce the risk of treatment failure. First, if doses are missed during the high-risk window, the immediate addition of another drug with a different mechanism of action from existing drugs may eliminate any low level of fully resistant mutants that has arisen. Alternatively, a patient could be treated preemptively using additional drugs during the entire high-risk period and switched to fewer drugs afterwards. Another important factor is the number of consecutively missed doses as shown previously [36]. Consecutively missed doses lead to exponential growth of ‘partially resistant’ mutants, and thus substantially increase the risk of *de novo* resistance.

Our theory also predicts the number of compensatory doses (*N*_*m*_) needed to compensate for missed doses, in order to eliminate preexisting mutants. Interestingly, clinical trials have shown that adherence levels tend to decrease over time [19,31]; we show that more doses are needed to compensate for missed doses that occur later in treatment because of the rebound of target cells. While many previous studies have focused on average adherence [18,19,29–31,36], we emphasize that the timing of the missed doses is also a critical determinant of treatment outcome and the risk of resistance.

There exist substantial heterogeneities among patients owing to variation in HCV genotypes, patient viral loads, death rates of infected cells [40,53] and effectiveness of drug penetration [47]. Our analysis has identified several factors that influence the impact of suboptimal adherence, particularly the rebound rate of target cells under treatment, the half-life of infected cells and the overall viral fitness, *R*_0_. We used the best available estimates of these parameters, but further empirical work is needed. If resistance profiles and viral parameters could be measured directly from a specific patient, then our framework linking these factors could be tailored to give patient-specific guidelines.

Certain model assumptions reflect uncertainties in our current knowledge of HCV infection. First, our prediction about time to viral extinction should be treated cautiously. We predict the time of extinction (as in other models [54–56]) by assuming that infected cells decline at a rate set by their death rate, and infection is cleared when the number of infected cells is below one. However, factors such as pressures from the immune system and infections in different tissue compartments may influence the extinction threshold. Furthermore, if DAA treatment causes intracellular viral RNA to decay with negligible replication [57], the decline of infected cells may result from a combination of cell recovery and death of infected cells. Indeed, sustained virological response has been observed in clinical trials of DAA combination therapies with shorter durations of treatment [5,6]. Our model can be adjusted easily once the decay dynamics of infected cells are understood better. Second, our model captures the main features of pharmacodynamics and viral dynamics by assuming quasi-equilibrium for viral populations and drug penetration into liver cells. Further work that incorporates detailed intracellular interactions [57] and different body compartments may improve model accuracy, once pertinent parameters are measured. However, a more detailed model may become analytically intractable.

This quantitative framework is a step towards developing a tool (for example, see Ref. [58]) for clinicians to design combination therapies and adaptively manage treatment regimens to achieve favorable clinical outcomes. It highlights the importance of characterizing resistance profiles of HCV, assessing readiness for treatment, and monitoring adherence patterns during treatment, so that treatment can be designed and adjusted in an evidence-based manner. This framework can be adapted easily to combination therapies based on interferon, entry inhibitors [52] or other DAA candidates, or treatments of other curable diseases without a latent reservoir.

## Materials and Methods

### HCV model and viral fitness in the presence of drug, R_eff_(t)

To analyze the dynamics of the virus, we constructed an ordinary differential equation (ODE) model to describe the long-term within-host dynamics of a single HCV strain under drug treatment, based on an established model developed by Neumann *et al*.[53] (see Supplementary Material). In the model, *ε* represents the proportion by which the therapy reduces viral growth (*ε* is in the range of 0 and 1). Then, the fitness of the virus, *R*_*eff*_(*t*), is the product of the complement of the therapy’s efficacy (1- *ε*(*τ*)), the reproductive number of the virus, *R*_0_, and the availability of target cells, *h*(*t*) (Eq 1).

### Average effective viral fitness when m doses are missed, R_ave,m_

To approximate the time-varying viral fitness, *R*_*eff*_(t), during the period when *m* consecutive doses are missed, we assume that the abundance of target cells stays constant. This is a good approximation, because the length of the period when consecutive doses are missed tends to be short compared to the time scale of target cell rebound. Then the only time-varying quantity in Eq 1 is *ε*(*τ*). We can calculate the average level of drug inhibition during the period when *m* doses are missed, *ε*_*ave*,*m*_, by incorporating parameters for pharmacokinetics and pharmacodynamics (for example, see Wahl and Nowak[36]). Then the time-average effective reproductive number, *R*_*ave*,*m*_(*t*), for a mutant when *m* consecutive doses are missed starting at time *t* can be expressed as Eq 2. In practice, because the precise number of target cells at time *t* is hard to estimate, we can approximate *R*_*ave*,*m*_(*t*) by setting *h*(*t*) = 1, and then *R*_*ave*,*m*_(*t*) becomes *R*_*ave*,*m*_(*t*) ≈ (1 – *ε*_*ave*,*m*_) ∙ *R*_0_. Because *h*(*t*)≤1, this always overestimates the viral fitness and thus is a conservative estimate in terms of guiding treatment. Note that the assumption that *h*(*t*) = 1 is valid only when the viral load at time *t* is much lower than it was before treatment, which is the case if adherence is not too low. Otherwise, *h*(*t*) would decrease significantly due to large amount of infection.

### The number of compensatory doses needed (N_m_)

To calculate *N*_*m*_ for each mutant, we make the simplifying assumption that the dynamics of the viral populations are at quasi-equilibrium, because changes in the viral populations occur much faster than changes in infected hepatocytes. Then, the dynamics of the number of cells infected by mutant viruses, *I*(*t*), are described by:

$$\frac{dI\left(t\right)}{d\left(t\right)}=\left(R_{eff}\left(t\right)-1\right)\cdot \delta \cdot I\left(t\right)$$ (5)

where *δ* is the death rate of infected hepatocytes. If we approximate *R*_*eff*_(*t*) using the constant *R*_*ave*,*m*_ for the period when doses are missed, Eq 5 can be solved analytically. Then, the number of infected cells after missing *m* consecutive doses starting at time *t*_0_ can be expressed as:

$$I\left(t_{0}+m+T\right)\approx I\left(t_{0}\right)\cdot \exp\left(\left(R_{ave,m}\left(t_{0}\right)-1\right)\cdot \delta \cdot m\cdot T\right)$$ (6)

We now consider the situation when *m* consecutive doses are missed, and ask how many uninterrupted doses (compensatory doses) must be taken so that the number of cells infected by the mutant is suppressed to a same number as if the *m* doses had not been missed. We first calculate the number of infected cells if the *m* consecutive doses are taken, i.e. if no doses is missed:

$$I_{optimal}\left(t_{0}+m\cdot T\right)\approx I\left(t_{0}\right)\cdot \exp\left(\left(R_{ave,0}\left(t_{0}\right)-1\right)\cdot \delta \cdot m\cdot T\right)$$ (7)

where *I*(*t*_0_) is the number of cells infected by the mutant at time *t*_0_, *R*_*ave*,0_ is the average effective reproductive number of the mutant when all doses are taken, and *T* is the scheduled interval between doses.

We then analyze the situation where a patient skips *m* consecutive doses, starting at time *t*_o_, and then takes *N*_*m*_ compensatory doses immediately afterwards. In this case, assuming the number of target cells does not change much during this period, we can approximate the number of cells infected by the mutant at the end of the *N*_*m*_ doses as:

$$I_{suboptimal}\left(t_{0}+m\cdot T+N_{m}\cdot T\right)\approx I\left(t_{0}\right)\cdot \exp\left(\left(R_{ave,m}\left(t_{0}\right)-1\right)\cdot \delta \cdot m\cdot T\right)\cdot I\left(t_{0}\right)\cdot \exp\left(\left(R_{ave,0}\left(t_{0}\right)-1\right)\cdot \delta \cdot N_{m}\cdot T\right)$$ (8)

By equating the right hand sides of Eqs 7 and 8 and solving the equation, we derive the expression for *N*_*m*_:

$$N_{m}\left(t_{0}\right)\approx \frac{R_{ave,m}\left(t_{0}\right)-R_{ave,0}\left(t_{0}\right)}{1-R_{ave,0}\left(t_{0}\right)}\cdot m$$ (9)

For potent therapies, usually *R*_*ave*,0_(*t*_0_) ≈ 0. Then we get Eq 3.

In the derivation above, we have assumed that the target cell abundance stays constant during the period under consideration. This would be a good approximation if only a few days of doses are missed or if the target cell has already rebounded to the infection-free level. If the abundance of target cells changes considerably during the period under consideration, an alternative, conservative approach would be to assume *h*(*t*) = 1 and take *N*_*m*,*max*_(*t*_0_) ≈ *m* ∙ (1 − *ε*_*ave*,*m*_) ∙ *R*_0_ compensatory doses after missing *m* consecutive doses of treatment.

### The number of doses to eradicate a mutant (N_erad_) and the number of cells infected by a mutant (I(t))

One important application of *N*_*m*_ is to predict the number of remaining doses needed to eradicate a mutant, *N*_*erad*_, in a patient during treatment. This number can be calculated as follows. If adherence is perfect, the number of infected cells declines exponentially at a rate set approximately by the death rate of infected cells, *δ*: (*t*) ≈ *I*_0_ ∙ exp(−*δ* ∙ *t*), where *I*_0_ is the number of cells infected by a mutant of interest before treatment. If we assume that a mutant goes extinct if the expected number of infected cells in a patient goes below 1, the number of doses needed to eradicate a mutant before treatment (assuming adherence is perfect), *N*_*erad*,0_, is calculated as: $N_{erad,0}\approx \frac{\log\left(I_{0}\right)}{\delta ∙T}$.

When doses are missed during treatment, it is clear from the calculation of *N*_*m*_ above that *N*_*m*_–*m* extra doses of treatment are needed to eradicate the virus. Therefore, if a patient has taken a total of *x* doses and has had *k* instances of missing doses before time *t*, with *m*_*i*_ days of doses missed in the *i* ^th^ instance (*i* = 1,2,…,*k*), then the number of remaining doses needed to eradicate the mutant is calculated as:

$$N_{erad}=N_{erad,0}-x+\sum_{i=1}^{k}\left(N_{m,i}-m_{i}\right)$$ (10)

We can use Eq 10 to predict the number of cells infected by a mutant as: *I*(*t*) ≈ exp(*δ* ∙ *N*_*erad*_(*t*) ∙ *T*). In our model, and a patient is cleared of infection when all mutants are driven to extinction. The accuracy of this approximation is shown in Figs 4D and 4F and 5D and 5F.

### The risk of full resistance if doses are missed (Φ_m_)

To calculate the risk of full resistance during the period when *m* doses are missed, we first calculate the number of cells newly infected by a partially resistant mutant when *m* doses are missed, Ω_*m*_(*t*). Again, we use *R*_*ave*,*m*_(*t*) to approximate *R*_*eff*_(*t*), the total number of cells infected by the mutant virus, starting at time *t*. Ω_*m*_(*t*) can be expressed as an integration of new infections during the period of missing doses (according to Eq 5):

$$\Omega_{m}\left(t\right)\approx R_{ave,m}\left(t\right)\cdot \delta \cdot \int_{t}^{t+m\cdot T}I\left(x\right)dx=I\left(t\right)\cdot \frac{R_{ave,m}\left(t\right)}{R_{ave,m}\left(t\right)-1}\cdot \left(e^{\left(R_{ave,m}\left(t\right)-1\right)\cdot \delta \cdot m\cdot T}-1\right)$$ (11)

The expected number of target cells that become infected by fully resistant mutant viruses, Φ_*m*_, is a product of the effective mutation rate from the partially resistant mutant to the fully resistant mutant (*μ*_*eff*_) and the total number of cells infected by the partially resistant mutant (Ω_*m*_): Φ_*m*_(*t*) = *μ*_*eff*_ ∙ Ω_*m*_(*t*), as shown in Eq 4.

Note that we track the population of newly infected cells to assess the risk of *de novo* generation of full resistance. This assumes implicitly that the fully resistant mutant is selected only when it enters a cell. This is a good assumption for DAAs that act on intracellular stages of the viral life-cycle, such as viral genome replication or assembly. However, in situations where the drug blocks viral entry into the cell, the mutant virus may have a selective advantage for entering a cell. Then the viral population should be tracked instead, but the results presented here still can be applied to drugs that block cell entry by multiplying with a simple scaling factor [59].

### Stochastic-deterministic hybrid simulation of multiple strains of HCV

We constructed a simulation model considering the dynamics of the baseline virus and all the potentially partially resistant mutants (see Supplementary Material). This simulation model follows a hybrid approach used previously to simulate the evolutionary dynamics of HIV [60]. It considers the dynamics of multiple strains of HCV deterministically (using ODEs) while treating the extinction and mutation processes as stochastic events (see Supplementary Material for detail).

In the simulation, a patient is treated for a total period of 24 weeks. We generate two types of dosing patterns: *random dosing* and *guided dosing*. For the *random dosing* pattern, doses are missed in blocks of 1–3 days at times chosen randomly with equal probability during the treatment period. This probability is set as a constant in each run, but varied across runs such that different overall levels of adherence are generated. In each simulation, we assume that at least one-day treatment is taken immediately after each dose-skipping event (i.e. 1, 2 or 3 consecutive missed doses), to ensure that two dose-skipping events do not occur consecutively (otherwise, longer blocks of doses would be missed than intended). For *guided dosing*, we ensure that doses are always taken during the high-risk window period predicted by our theory. After this high-risk window period, we set a constant probability of missing doses in blocks of 1–3 days. Immediately after a block of doses is missed, we ensure a sufficient number of uninterrupted doses (calculated as N_*m*_) are always taken. If the virus is not eradicated after the 24-week treatment period, the patient is treated with an uninterrupted number of doses as predicted by our theory. The outcome of the simulation at the end of the procedure is reported.

## Supporting Information

S1 Text Supplementary materials. (DOCX)

S1 Fig Sensitivity analysis of the risk of *de novo* resistance to variations of key parameter values, R_0,mut_ (panels A,B), δ (panel C) and the rate of recovery of target cells upon treatment (panel D). In each panel, the trajectories show how the risk of *de novo* resistance (Log_10_Φ_m_) changes over time if adherence is perfect. Figures are plotted using the same parameter settings as trajectories ‘a’ in Fig 3 in the main text, except that R_0,mut_ = 5 in panel A, R_0,mut_ = 15 in panel B, δ = 0.5 in panel C and α = 1.95*10^4^, *d* = 0.015 in panel D. In the main results, i.e. Fig 3, the parameter values used are R_0,mut_ = 10, δ = 0.15, λ = 1.95*10^5^, *d* = 0.15.
(TIFF)

S2 Fig The predicted number of additional days of doses needed to compensate for the first instance of missing 1 (green lines), 2 (blue lines) or 3 (red lines) consecutive days of doses (maximum N_m_ for all partially resistant mutants), and the high-risk window period of *de novo* resistance (shaded area; Φ_m_>0.01 for any of the partially resistant mutants as shown in Fig 4B). **(A)** Predictions for patients with the wild-type virus at baseline before treatment. The areas below the curves are white, indicating that the risk of *de novo* resistance is always low. **(B)** Predictions for patients with the Y93H mutant virus at baseline. The initial increases of the number of days of compensating doses are due to the increase of the number of target cells upon treatment, and the sudden drops during later periods of treatment are due to the elimination of particular partially resistant mutant lineages. Note that these curves are calculated under the assumption that adherence is perfect except for the 1–3 days of missed doses being considered, i.e. it is a prediction for the first instance of missed doses. For cases where multiple instances of missed doses have occurred, one needs to calculate the values of N_m_ and Φ_m_ for each mutant based on the adherence pattern, and then integrate them together by choosing the highest values of N_m_ and Φ_m_ for those mutants.
(TIFF)

S3 Fig Theory correctly predicts the number of cells infected by two mutants (L31V and L31M+Y93H) generated in the hybrid model simulation when doses are missed randomly (panels A,B) or guided by adaptive treatment theory (panels C,D) for patients with the wild-type virus at baseline. L31V and L31M+Y93H are the two most likely mutants that generate full resistance. The axes are the theory prediction (x-axis) and model simulation (y-axis) of the Log_10_ of the number of mutants, which are calculated as the cumulative numbers of Log_10_ Φ _m_(*t*)/μ _*mut*_ for all missed doses.
(TIFF)

S4 Fig The theory correctly predicts the number of cells infected by mutant viruses at the end of 24-weeks’ treatment in the hybrid model simulation when adherence is greater than 70% (vertical dashed lines) and doses are missed randomly, for patients with the Y93H mutant virus at baseline. The y-axis shows the log_10_ difference between the theory prediction and the model simulation at the end of the 24-weeks’ treatment. Note that when adherence is lower than 70%, the population of infected cells grows to high levels close to the pre-treatment level, where further growth is curtailed by target cell limitation. As a result, the theoretical prediction overestimates the number of cells infected by the mutant virus significantly because we assume the number of target cells is not limited.
(TIFF)

S5 Fig Comparison between theory prediction and simulation of the numbers cells infected by three mutants (L31M+Y93H, L31V+Y93H, L31V+Q54H+Y93H) generated when doses are missed randomly, for patients with the Y93H mutant virus at baseline. **(A,B,C)** The axes are the theory prediction (x-axis) and model simulation (y-axis) of the Log_10_ of the number of cells infected by different mutants, which are calculated as the cumulative numbers of Log_10_ Φ_m_/μ_*mut*_ for all missed doses (L31M+Y93H in panel A; L31V+Y93H in panel B; L31V+Q54H+Y93H in panel C). **(D,E,F)** Our theory prediction is accurate for adherence greater than 70%, but overestimates the number of cells infected by the mutant virus significantly when adherence is lower than 70%, for the same reason as explained in the legend of S4 Fig.
(TIFF)

S6 Fig Theory correctly predicts the number of cells infected by three mutants (L31M+Y93H, L31V+Y93H, L31V+Q54H+Y93H) generated in the hybrid model simulation when doses are guided by adaptive treatment theory, for patients with the Y93H virus at baseline. **(A,B,C)** The axes are the theory prediction (x-axis) and model simulation (y-axis) of the Log_10_ of the number of mutants (L31M+Y93H in panel A; L31V+Y93H in panel B; L31V+Q54H+Y93H in panel C), which are calculated as the cumulative numbers of Log_10_ Φ_m_(*t*)/ μ_*mut*_ for all missed doses. **(D,E,F)** the Log_10_ differences between theory prediction and model simulation as shown in panels (A,B,C). Note that our theory agrees very well for mutants L31M+Y93H and L31V+Y93H. For mutant L31V+Q54H+Y93H, the stochastic extinction and appearance of this mutant generates stochastic deviations of the simulation from theory prediction.
(TIFF)

S7 Fig The impact of lower viral fitness (R_0_ = 5) on treatment outcomes and adaptive treatment strategies of combination therapy with daclatasvir and asunaprevir, with the wild-type virus at baseline. Panels A-F show the same plots as Fig 4 in the main text, except that the fitness parameter R_0_ for the wild-type virus is assumed to be 5. The treatment outcome improves for all scenarios for this lower viral fitness (compare with Fig 4 in the main text). Using the adaptive treatment strategy prevents viral relapse and *de novo* resistance if overall adherence is greater than 60% (panel E). Panel F is empty because all patients are cleared of infection after 24 weeks.
(TIFF)

S8 Fig The impact of lower viral fitness (R_0_ = 5) on treatment outcomes and adaptive treatment strategies of combination therapy with daclatasvir and asunaprevir, with the Y93H virus at baseline. Panels A-F show the same plots as Fig 5 in the main text, except that the fitness parameter R_0_ for the wild-type virus is assumed to be 5. The treatment outcome improves for all scenarios for this lower viral fitness (compare with Fig 5 in the main text). Using adaptive treatment strategy reduced the risk of *de novo* resistance (panels E). Our theory correctly predicts the number of infected cells in a patient at the end of 24 weeks’ treatment.
(TIFF)

S9 Fig The impact of higher viral fitness (R_0_ = 15) on treatment outcomes and adaptive treatment strategies of combination therapy with daclatasvir and asunaprevir, with the wild-type virus at baseline. Panels A-F show the same plots as Fig 4 in the main text, except that the fitness parameter R_0_ for the wild-type virus is assumed to be 15. The treatment outcome improves for all scenarios for this lower viral fitness (compare with Fig 4 in the main text). Our theory correctly predicts the number of infected cells in a patient at the end of 24 weeks’ treatment.
(TIFF)

S10 Fig The impact of higher viral fitness (R_0_ = 15) on the treatment outcomes and adaptive treatment strategies of combination therapy of daclatasvir and asunaprevir with the Y93H virus at baseline. Panels A-F show the same plots with Fig 5 except that in the analytical derivation and model simulation, the fitness parameter R_0_ for the Y93H mutant virus is assumed to be 15. The risks of viral relapse and *de novo* resistance become higher when the viral fitness, R_0_, is higher. Using adaptive treatment strategy can prevent *de novo* resistance and improve treatment outcomes (panels E and F). Our theory correctly predicts the number of infected cells in a patient at the end of 24 weeks’ treatment when doses are guided (panels F). Our theory does not predict the number of infected cells at the end of treatment well, when doses are missed randomly and the adherence is low. This is because, when adherence is low, the viral load often rebounds back to the pre-treatment level, where it is limited by target cell availability. This phenomenon is not included in our theory, which overestimates the number of viruses as a result.
(TIFF)

S11 Fig The impact of higher viral clearance rate (*δ* = 0.5) on the treatment outcomes and adaptive treatment strategies of combination therapy of daclatasvir and asunaprevir with the wild-type genotype 1b virus at baseline. Panels A-F show the same plots with Fig 4 except that in the analytical derivation and model simulation, the viral clearance rate, **δ**, is assumed to be 0.5 instead of 0.15 in Fig 4 (but note that R_0_ for the viruses is kept the same). When the viral clearance rate increases, it takes less time to eradicate the virus from a patient. However, when doses are missed, the population of mutant viruses expands more quickly, because the half-life of the infected cells is shorter and thus it undergoes a higher number of replication generations during the period of missed doses. Using the adaptive treatment strategy can prevent viral relapse and *de novo* resistance and improve treatment outcome (panels E). Our theory correctly predicts the number of infected cells in a patient at the end of 24 weeks’ treatment when doses are guided (panel D).
(TIFF)

S12 Fig The impact of higher viral clearance rate (*δ* = 0.5) on the treatment outcomes and adaptive treatment strategies of combination therapy of daclatasvir and asunaprevir with the Y93H virus at baseline. Panels A-F show the same plots with Fig 5 except that in the analytical derivation and model simulation, we assume the viral clearance rate, **δ**, is 0.5 instead of 0.15 (but note that R_0_ for the viruses is kept the same). As seen in S11 Fig for the scenario with the wild-type virus at baseline, it takes less time to eradicate the virus from a patient for this higher viral clearance rate. However, when doses are missed, the population of mutant viruses expands more quickly, increasing the risk of viral relapse and *de novo* resistance. Using adaptive treatment strategy can prevent viral relapse, *de novo* resistance and improve treatment outcome (panels E and F). Our theory does not predict the number of infected cells at the end of treatment well, when doses are missed randomly and adherence is low. This is because, during the time period when doses are missed, the rebound of the viruses is quicker when **δ** is higher (because the viral generation time is shorter). When adherence is low, the viral load often rebounds back to the pre-treatment level, where it is limited by target cell availability. This phenomenon is not included in our theory, which overestimates the number of viruses as a result.
(TIFF)
