# Supplementary material for: Rational Design and Adaptive Management of Combination Therapies for Hepatitis C Virus Infection
Source: PLoS Comput Biol. 2015 Jun 30;11(6):e1004040. doi: 10.1371/journal.pcbi.1004040 (PMC4488346; doi:10.1371/journal.pcbi.1004040)
Supplement: S1 Text — (DOCX) [file pcbi.1004040.s001.docx]

# Text S1 - Supplementary materials for

## **Rational design and adaptive management of combination therapies for Hepatitis C virus infection**

Ruian Ke, Claude Loverdo, Hangfei Qi, Ren Sun, James O. Lloyd-Smith

Table of Contents

Supplementary Text 2

1. HCV model and derivation of viral fitness (R_0_) 2

2. Combination therapy of daclatasvir and asunaprevir 3

3. Simulation of a hybrid multi-strain model 8

4. Sensitivity analysis 9

Sensitivity of predicted clinical outcomes to changes in parameter values 9

Robustness of adaptive treatment strategy to variations in parameter values 11

Supplementary References: 12

# Supplementary Text

## HCV model and derivation of viral fitness (R_0_)

We first construct an ordinary differential equation (ODE) model to describe the long-term within-host dynamics of a single HCV strain under drug treatment. This model is based on an established model developed by Neumann *et al.*[[1](#_ENREF_1)]. It considers the dynamics of the target hepatocytes (H), the infected hepatocytes (I) and the HCV viruses (V). Guedj *et al.* have recently shown that the dynamics of viral loads during the first few days of treatment with direct acting antivirals (DAAs) are better described by a multi-scale model that considers intracellular dynamics of HCV RNAs[[2](#_ENREF_2)]. However, since here we are mostly interested in the longer-term dynamics of HCV infection, stretching for weeks or months, the simpler model by Neumann *et al.* is a good approximation.

The ODE model describing the system is:

| $\frac{dH}{dt}=\lambda-d\cdot H-\beta\cdot H\cdot V$ $\frac{dI}{dt}=\beta\cdot H\cdot V-\delta\cdot I$ $\frac{dV}{dt}=\left( 1-\varepsilon\right)\cdot p\cdot I-c\cdot V$ | (S1) |
| --- | --- |

Uninfected target hepatocytes are produced at constant rate, *λ*, and cleared at per capita rate *d*. The infection rate for uninfected hepatocytes is proportional to V, with infection rate constant *β*.The per capita death rate of infected hepatocytes is *δ*. The virions are cleared at per capita rate *c*. In the absence of drug treatment, viruses are produced from infected hepatocytes at rate *p*. Under drug treatment, the production of viruses from infected cells is reduced to rate (1-*ε*)**p*, where *ε* is the efficacy of the drug of drug treatment.

It has been shown that the number of target hepatocytes increases quickly after initiation of effective treatment [[3](#_ENREF_3)]. In our model, this rebound rate is determined by the parameters *λ* and *d*, and the number of target hepatocytes in the absence of HCV infection, *H*_0_, is determined by the ratio of this two parameters, i.e. *H*_0_=*λ/d*. We chose values of *λ* and *d* such that the target hepatocytes increase on a similar timescale to the results of Rong *et al.*[[3](#_ENREF_3)], while keeping the total number of target hepatocytes constant in the absence of infection. To calculate *R_eff_*(*t*) (in the main text), we set *h*(*t*) as *h*(*t*)=*H*(*t*)/*H*­_0_, i.e. the normalized abundance of target cells.

Based on Eqns. S1, we can calculate the reproductive number, *R*_0_, in the absence of drug, as:

$R_{0}={H_{0}\cdot\beta\cdot p}/{(\delta\cdot c)}$ (S2)

We set *R*_0_=10 in our main analysis, and this choice is in agreement with previous studies[[1](#_ENREF_1),[3](#_ENREF_3)].

We assume there are 2*10^11^ hepatocytes in an infected liver[[3](#_ENREF_3)]. It has been shown that 1%-50% of all hepatocytes are infected in chronically infected patient[[4](#_ENREF_4),[5](#_ENREF_5)]. Thus, we assume that only half of the total hepatocytes can potentially be infected in the absence of treatment, i.e. H_0_=1*10^11^, as in Rong *et al.*[[3](#_ENREF_3)]. The value of *p* is set such that the reproductive number *R*_0_ of the virus in the absence of drug is 10 (calculated above in Eqn. S2). The parameter values are listed in Table 1.

**Table 1. Parameter values in the HCV model.**

| **Parameters** | **Values** | **Unit** | **References** |
| --- | --- | --- | --- |
| **λ** | 1.0*10^6^ | cells ml^-1^ day^-1^ | See text |
| **d** | 0.15 | day^-1^ | See text |
| **β** | 8.88*10^-8^ | ml day^-1^ | Rong *et al.*[[3](#_ENREF_3)] |
| **δ** | 0.15 | day^-1^ | Neumann *et al.*[[1](#_ENREF_1)] |
| **p** | 56.5 | day^-1^ | See text |
| **c** | 22.3 | day^-1^ | Guedj *et al.*[[2](#_ENREF_2)] |

## Combination therapy of daclatasvir and asunaprevir

**Pharmacokinetics/Pharmacodynamics**

In general, pharmacokinetics of HCV DAAs follow a characteristic pattern: drug concentration increases quickly to a peak level after dosing and then decreases exponentially until the next dose is administered. We use three pharmacokinetic parameters to describe this pattern: the time to reach peak concentration after taking the drug (*τ*), the peak drug concentration (*C_max_*) and the minimum drug concentration before the next treatment (*C_min_*). We assume the active drug concentrations in the liver are related to the drug concentration in the plasma (where data are measured) by a constant ratio $\eta$. Then, the active tissue concentration of a drug, *C*(*t*), between dosing intervals can be described using the following equation:

$C(t)=\left\{ \begin{matrix} (C_{min}+\frac{C_{max}-C_{min}}{\tau}\cdot t)\cdot\eta& 0<t<\tau\\ C_{max}\cdot exp(-w\cdot\left( t-\tau\right))\cdot\eta& \tau<t<T \end{matrix} \right.$ (S3)

where *C_max_* and *C_min_* are the maximum and minimum drug concentrations in the plasma, *T* is the interval between two consecutive doses, and $w=\frac{1}{T-\tau}\cdot log \frac{C_{min}}{C_{max}}$ . The value of *w* is calculated such that the drug concentration at the beginning is equal to the concentration at the end of a single dose.

The regimen used in clinical trials for the combination therapy of daclatasvir and asunaprevir is 60mg once daily for daclatasvir and 200mg twice daily for asunaprevir[[6](#_ENREF_6),[7](#_ENREF_7)]. The value of liver-to-plasma ratio, $\eta$, for daclatasvir is set as $\eta=0.094$ for daclatasvir as shown in recent work[[8](#_ENREF_8)]. The value of liver-to-plasma ratio for asunaprevir in human subject is still not clear, although this drug has been shown to have a large liver-to-plasma ratio in animal models[[9](#_ENREF_9)]. Clinical data from treated patients shows that mutant Q80L+D168V has EC_90_(the drug concentration suppress 90% production) of 55 nM, and it is resistant to asunaprevir treatment in a patient (PT-29) with trough plasma concentration of 18-33nM[[10](#_ENREF_10)]. This suggests that the tissue concentration of asunaprevir in the liver is at a similar level to its level in the plasma, and therefore, we have set $\eta=1.0$ for asunaprevir. The parameters used for the pharmacokinetics of these two drugs are shown in Table 2.

**Table 2. Pharmocokinetic parameter values for daclatasvir and asunaprevir treatment used in the simulation model.**

| **Parameter** | **Daclatasvir** **[**[**11**](#_ENREF_11)**]**  **60mg QD** | **Asunaprevir [**[**12**](#_ENREF_12)**]**  **200mg BID** |
| --- | --- | --- |
| C_max_ | 1726 ng/ml | 268 ng/ml |
| C_min_ | 255 ng/ml | 35 ng/ml |
| T | 1 day | 0.5 day |
| τ | 1.5 hour | 3.0 hour |
| $\eta$ | 0.094 | 1 |

Since daclatasvir and asunaprevir act independently on NS5A and NS3 genes, here in the model, we calculate the inhibition of viral growth using Bliss independence[[13](#_ENREF_13)]:

$\varepsilon_{i}(t)=\frac{1}{\left( 1+\frac{C_{dac}(t)}{{EC}_{50\_dac}} \right)\cdot\left( 1+\frac{C_{asu}(t)}{{EC}_{50\_asu}} \right)}$ (S4)

where *C_aac_*(t) and *C_asu_*(t) are the active tissue concentrations of daclatasvir and asunaprevir, respectively, and *EC*_50_*__dac_* and *EC*_50_*__asu_* are the corresponding EC_50_ values of for the viral strain under consideration. The average inhibition during the period when *m* doses are missed, $\varepsilon_{ave,m}$, is calculated numerically using Eqns. S3 and S4. Another model for pharmacological independence is Loewe independence[[14](#_ENREF_14)]. Changing the model to Loewe independence slightly changes our prediction about the fitness of each mutant under treatment, but does not alter the conclusion of the model.

**Characterizing preexisting mutants (PMs) and predicting the time needed to eradicate the PMs**

We first approximate the equilibrium level of cells infected by the baseline virus. The baseline virus is the viral strain that dominates the population before treatment, i.e. either the wild-type or the Y93H mutant in this study. Its equilibrium abundance before treatment can be derived from the single strain model in Eqn. S1, yielding:

$$I_{0}=\frac{\lambda}{\delta}-\frac{c\cdot d}{\beta\cdot p}$$

Under effective treatment, the population of infected cells declines exponentially, at a rate set by the half-life of the infected cell (1/*δ*). Then, the time needed to eradicate the baseline virus under perfect adherence can be calculated as:

$t_{erad,base}=\left( \log I_{0}-\log I_{ext} \right)\cdot\frac{1}{\delta}=\left( \log(\frac{\lambda}{\delta}-\frac{d\cdot c}{\beta\cdot p})-\log I_{ext} \right)\cdot\frac{1}{\delta}$ (S5)

where *I_ext_* is the extinction threshold of infected cells below which the virus goes extinct. In our model, we set I_ext_=1/15000 copy/ml (assuming there are 15L of extracellular fluid[[3](#_ENREF_3)]).

If the fitness of a mutant relative to the wild-type is 1-*s_mut_*, then the frequency of resistant mutant virus before treatment can be approximated as[[15](#_ENREF_15)]:

$$I_{mut}=\frac{\mu_{mut}}{s_{mut}}\cdot I_{0}$$

where *μ_mut_* is the mutation rate from the baseline virus to the mutant. Then the time needed to eradicate the mutant virus*, t_erad,mut_*, can be calculated as:

$t_{erad,mut}=\left( \log I_{mut}-\log I_{ext} \right)\cdot\frac{1}{\delta}=\left( \log(\frac{\mu_{mut}}{s_{mut}}\cdot I_{0})-\log I_{ext} \right)\cdot\frac{1}{\delta}$ (S6)

In the model, the mutation rates are set as 2.85*10^-5^ and 1.5*10^-6^ per infection cycle in infected cells for transitions and transversions, respectively (Loverdo et al, unpublished work). A mutant is considered as a preexisting mutant if the number of infected cells calculated for the mutant is above 1 copy in a patient, which corresponds to 1/15,000 copy/ml[[3](#_ENREF_3)]. If a mutant strain has a higher replicative fitness than the wild-type virus (as measured in the replicon system), we set its relative fitness to 0.99 to ensure that the baseline virus is the dominant strain before treatment. This assumption is required by definition of the baseline strain, and reflects possible differences between fitnesses measured in replicon systems and *in vivo*.

**Characterizing fully resistant mutants**

Since daclatasvir and asunaprevir act independently on different target genes (NS5A and NS3, respectively), we define mutant viruses bearing resistance mutations, i.e. mutants show positive growth under therapy, against both daclatasvir and asunaprevir as potentially fully-resistant to the combination therapy.

To characterize these mutants, we first find mutations that cause resistance, i.e. positive growth, under monotherapies of daclatasvir or asunaprevir, and we assume that daclatasvir and asunaprevir concentrations are the same with the corresponding concentrations in the combination therapy. For each mutant that have been reported to show higher resistance level than the wild-type, we calculated the effective reproductive number under daclatasvir and asunaprevir monotherapies (*R_eff,dac_max_* and *R_eff,asu_max_*, respectively) when the target cell population is at its infection-free level (Table 3 and 4). We find that, if the wild-type virus is the baseline strain before treatment, the preexisting mutants that are resistant to daclatasvir monotherapy are L31M/V+Y93H, and the preexisting mutants resistant to asunaprevir monotherapy are D168A/V (Table 3). Then, combinations of these mutations are potentially fully resistant mutants, e.g. L31V+Y93H+D168V. If the Y93H mutant virus is the baseline strain before treatment, the preexisting mutants that are resistant to daclatasvir monotherapy are L31M/V+Y93H and L31V+Q54H+Y93H, and the preexisting mutants that are resistant to asunaprevir monotherapy is the same with the case when the wild-type virus is at baseline, i.e. D168A/V (Table 4).

**Table 3. The resistance profile of genotype 1b mutants when the wild-type virus is at baseline.**

| Mutant† | EC_50_ to daclatasvir treatment* | EC_50_ to asunaprevir treatment* | Relative replication (1-*s_mut_*) * | *R_eff,dac_max_* | *R_eff,asu_max_* |
| --- | --- | --- | --- | --- | --- |
| **WT** | 0.0026 | 0.86 | 1.0 | 0.0004 | 0.0716 |
| **L31M** | 0.0084 | 0.86 | 0.99** | 0.0011 | 0.0708 |
| **L31V** | 0.0716 | 0.86 | 0.99** | 0.0096 | 0.0708 |
| **L31W** | 0.2100 | 0.86 | 0.99** | 0.0281 | 0.0708 |
| **Q54H** | 0.0032 | 0.86 | 0.83 | 0.0004 | 0.0594 |
| **Y93H** | 0.0621 | 0.86 | 0.27 | 0.0023 | 0.0193 |
| **D168A** | 0.0026 | 109 | 0.37 | 0.0001 | **1.6253** |
| **D168V** | 0.0026 | 241 | 0.29 | 0.0001 | **1.7968** |
| **L31F+Y93H** | 14.87 | 0.86 | 0.29 | 0.4673 | 0.0208 |
| **L31M+Y93H** | 18.23 | 0.86 | 0.70 | **1.3248** | 0.0501 |
| **L31V+Y93H** | 37.94 | 0.86 | 0.50 | **1.5923** | 0.0358 |
| Q54H+Y93H | 0.0243 | 0.86 | 0.22 | 0.0007 | 0.0157 |
| L31V+Q54H+Y93H | 48.74 | 0.86 | 0.99** | 3.6766 | 0.0708 |

† Bold names denote mutants that are preexisting in a patient.

* Data taken from Fridell et al.[[16](#_ENREF_16)] and McPhee et al.[[17](#_ENREF_17)].

** The fitness values of these mutants relative to the wild-type are set to 0.99, because the values measured in the replicon system were higher than 1 .

**Table 4. The resistance profile of genotype 1b mutants when the Y93H virus is at baseline.**

| Mutant† | EC_50_ to daclatasvir treatment* | EC_50_ to asunaprevir treatment * | Relative replication  (1-*s_mut_*) * | *R_eff,dac_max_* | *R_eff,asu_max_* |
| --- | --- | --- | --- | --- | --- |
| **Baseline-Y93H**** | 0.0621 | 0.86 | 1.0 | 0.0084 | 0.0716 |
| **L31F+Y93H** | 14.87 | 0.86 | 0.29 | 0.4673 | 0.0208 |
| **L31M+Y93H** | 18.225 | 0.86 | 0.70 | **1.3248** | 0.0501 |
| **L31V+Y93H** | 37.935 | 0.86 | 0.50 | **1.5923** | 0.0358 |
| **Q54H+Y93H** | 0.0243 | 0.86 | 0.22 | 0.0007 | 0.0157 |
| **Y93H+D168A** | 0.0621 | 109 | 0.37 | 0.0031 | **1.6253** |
| **Y93H+D168V** | 0.0621 | 241 | 0.29 | 0.0024 | **1.7968** |
| **L31V+Q54H+Y93H** | 48.735 | 0.86 | 0.99*** | **3.6766** | 0.0708 |

† Bold names denote mutants that are preexisting in a patient.

* Data taken from Fridell et al.[[16](#_ENREF_16)] and McPhee et al.[[17](#_ENREF_17)].

** We assume that the baseline virus, Y93H mutant, has the highest fitness, set its relative fitness as 1.0.

*** The fitness value of this mutant relative to the wild-type is set to 0.99, because the value measured in the replicon system is higher than 1.

## Simulation of a hybrid multi-strain model

We construct a simulation model considering the dynamics of the baseline virus and all the preexisting mutants that are shown in Table 3 and 4. This simulation model follows a hybrid approach used previously for simulating HIV evolutionary dynamics[[18](#_ENREF_18)]. The model considers the dynamics of multiple strains of HCV deterministically, using ODEs, while treating the extinction and generation of mutants as stochastic events. The model tracks the population of uninfected and infected hepatocytes. Since the dynamics of viruses are much quicker than those of infected cells, we assume that the virus population is at quasi-equilibrium with respect to the dynamics of the infected cell population: the viral abundance is then given by $V\left( t \right)=\left( 1-\varepsilon\right)\cdot p\cdot I(t)/c$. Then, the ODEs describing the dynamics of the multi-strain system become:

| $\frac{dH}{dt}=\lambda-d\cdot H-\frac{\beta\cdot p}{c}\cdot H\cdot\sum_{i=1}^{n} \left( 1-\varepsilon_{i} \right)\cdot I_{i}$  $\frac{dI_{i}}{dt}=\frac{\beta\cdot p}{c}\cdot H\cdot\left( 1-\varepsilon_{i} \right)\cdot I_{i}-\delta\cdot I_{i}$ | (S7) |
| --- | --- |

where *H* is the concentration of target hepatocytes, and *I_i_* is the concentration of hepatocytes infected by viral strain *i*.

The mutation process is treated stochastically. During the simulation, the ODEs are first simulated for a fixed time increment ($\Delta$*t*=0.01 day). At the end of each time increment, we approximate the number of cells newly infected by viruses from cells infected by the i^th^ strain as $\frac{\beta\cdot p}{c}\cdot H\cdot\left( 1-\varepsilon_{i} \right)\cdot I_{i}\cdot L\cdot\Delta t$, where *L* is the total volume of the liver. Of these newly infected cells, the number of cells in which the infecting viral lineage mutates from the i^th^ strain to the j^th^ strain can be drawn from a binomial distribution with probability of $u_{ij}$, which is the mutation rate from the i^th^ to the j^th^ strain. We then convert the number to concentration by dividing the number by *L*:

$${\Delta I}_{i,j}=B(\frac{\beta\cdot p}{c}\cdot H\cdot\left( 1-\varepsilon_{i} \right)\cdot I_{i}\cdot L\cdot\Delta t,u_{ij})/L$$

Each time step, the concentration of each strain is updated according to the values of ${\Delta I}_{i,j}$. We then check the number of cells infected by each strain. If the number is less than 1 copy per individual, we set it to 0 in the system, i.e. extinction. These procedures are iterated until all infected cells are extinct or simulation time exceeds 24 weeks (for random dosing pattern) or guided dosing period (for guided dosing).

The dosing pattern is generated according to the procedure described in Online Methods. Once the dosing pattern is generated, the drug concentrations are calculated according to Eqn. S3.

## Sensitivity analysis

In the analytical derivations, the parameters that determine the values of N_m_ and Φ_m_ are: the rate at which target hepatocytes become available under treatment (set by the values of parameters *λ* and *d*); the overall viral fitness, *R*_0_; and the clearance rate of infected hepatocytes, *δ*. We performed two rounds of sensitivity analysis, first testing how changes in these parameter values impact the clinical outcomes predicted by our theory, and then testing the robustness of our adaptive treatment strategy to changes in these parameter values.

### Sensitivity of predicted clinical outcomes to changes in parameter values

**The overall viral fitness, *R*_0_**

The viral fitness parameter, *R*_0_, has several impacts on the predictions of the model. To evaluate the impact of changes in *R*_0_ on the time needed to eradicate the virus, we can substitute the expression of *R*_0_ into Eqn. S5 as:

$t_{erad,base}=\left( \log(\frac{\lambda}{\delta}\cdot\left( 1-\frac{1}{R_{0}} \right))-\log I_{ext} \right)\cdot\frac{1}{\delta}$ (S8)

From this equation it can be seen that, if *λ* and *δ* are kept constant, higher R_0_ leads to a higher level of infected hepatocytes before treatment, and thus, a longer time needed to eradicate the virus. However, this increase may be small, because *t_erad,base_* changes in proportion to the logarithm of $1-\frac{1}{R_{0}}$.

The value of N_m_ scales linearly with *R*­_0_ (Eqn. 2 in the main text). Thus, the higher the value of *R*_0_, the more doses needed to compensate for any missed doses.

The value of Φ_m_ increases almost exponentially with an increase in *R*_0_ (Eqn. 3 in the main text). Therefore the risk of generating fully-resistant mutants increases drastically as *R*_0_ rises (see Fig. S1a,b). This is because the viral population of a mutant increases exponentially if *R_eff,m_* becomes greater than 1, which leads to an exponential increase in the risk of generating fully resistant mutants.

**The clearance rate of infected hepatocytes, *δ***

The clearance rate of infected hepatocytes, *δ*, influences our theoretical predictions in two ways. First, the time needed to eradicate the virus depends linearly on the inverse of the clearance rate, 1/*δ* (Eqn. S5 and S6). The quicker the clearance rate of infected hepatoctyes, the shorter time needed to eradicate the virus. Second, changes in the value of *δ* affect the risk of generating fully resistant mutants when doses are missed (Eqn. 3 in the main text). If we let the fitness of a virus, *R*_0_, unaffected by changes of *δ*, larger values of *δ* lead to shorter half-lives of infected hepatocytes, and higher risk of generating fully-resistant mutants, because the viral lineage undergoes more rounds of replication during a fixed dosing period (Fig. S1c).

**The rate at which target hepatocytes become available under treatment**

N_m_ is linearly dependent on the level of target hepatocytes (Eqn.2 in the main text), and thus, slower rebound of target hepatocytes would decrease the number of compensatory doses needed if doses are missed before the target hepatocyte population rebounds back to its infection-free level.

The rebound rate of target hepatocytes also impacts Φ_m_, through its influence on the effective viral fitness, *R_eff,m_* (Eqn. 3 in the main text). In our model, the rebound rate is set by the values of parameters *λ* and *d*. If we keep the number of hepatocytes before treatment constant, by keeping the ratio of *λ* over *d* constant, then a slower rebound rate (lower value of both *λ* and *d*) results in a substantially reduced rate of generating *de novo* resistance (Fig. S1d).

### Robustness of adaptive treatment strategy to variations in parameter values

We first tested the robustness of adaptive treatment strategy to lower or higher values of R_0_ (*R*_0_=5 and *R*_0_=15, as opposed to *R*_0_=10 for our main results). When *R*_0_=5, both the number of compensatory doses (N_m_) and the potential to generate *de novo* resistance (Φ_m_) decrease substantially, as predicted by our model (Fig. S6-S7). This leads to a shorter high-risk window period when mutant Y93H is the baseline strain (Fig. S7) and lower adherence levels are required to eradicate the virus. When *R*_0_=15, we observe the opposite pattern: higher adherence levels are required to eradicate the virus and there is a longer high-risk window period when mutant Y93H is the baseline strain (Fig. S8-S9).

We then tested how well our adaptive treatment strategy works when the half-life of infected hepatocytes is shorter. As shown in Fig. S10 and S11, the time needed to eradicate the virus decreases substantially, to 12 weeks of effective treatment. Our adaptive treatment strategy improves clinical outcome especially when Y93H mutant is at the baseline (Fig. S11).

In general, we find our adaptive treatment strategy is robust against variations in key parameter values. Under all parameter values tested, the adaptive treatment strategy delivers substantially better patient outcomes than random dosing with the same overall adherence levels.

# Supplementary References:

1. Neumann AU, Lam NP, Dahari H, Gretch DR, Wiley TE, et al. (1998) Hepatitis C viral dynamics in vivo and the antiviral efficacy of interferon-alpha therapy. Science 282: 103-107.

2. Guedj J, Dahari H, Rong LB, Sansone ND, Nettles RE, et al. (2013) Modeling shows that the NS5A inhibitor daclatasvir has two modes of action and yields a shorter estimate of the hepatitis C virus half-life. Proceedings of the National Academy of Sciences of the United States of America 110: 3991-3996.

3. Rong L, Dahari H, Ribeiro RM, Perelson AS (2010) Rapid emergence of protease inhibitor resistance in hepatitis C virus. Sci Transl Med 2: 30ra32.

4. Wieland S, Makowska Z, Campana B, Calabrese D, Dill MT, et al. (2014) Simultaneous detection of hepatitis C virus and interferon stimulated gene expression in infected human liver. Hepatology 59: 2121-2130.

5. Liang Y, Shilagard T, Xiao SY, Snyder N, Lau D, et al. (2009) Visualizing hepatitis C virus infections in human liver by two-photon microscopy. Gastroenterology 137: 1448-1458.

6. Pol S, Ghalib RH, Rustgi VK, Martorell C, Everson GT, et al. (2012) Daclatasvir for previously untreated chronic hepatitis C genotype-1 infection: a randomised, parallel-group, double-blind, placebo-controlled, dose-finding, phase 2a trial. Lancet Infect Dis 12: 671-677.

7. Lok AS, Gardiner DF, Lawitz E, Martorell C, Everson GT, et al. (2012) Preliminary study of two antiviral agents for hepatitis C genotype 1. N Engl J Med 366: 216-224.

8. Ke R, Loverdo C, Qi H, Olson CA, Wu NC, et al. (2014) Modelling clinical data shows active tissue concentration of daclatasvir is 10-fold lower than its plasma concentration. J Antimicrob Chemother 69: 724-727.

9. McPhee F, Sheaffer AK, Friborg J, Hernandez D, Falk P, et al. (2012) Preclinical Profile and Characterization of the Hepatitis C Virus NS3 Protease Inhibitor Asunaprevir (BMS-650032). Antimicrob Agents Chemother 56: 5387-5396.

10. Karino Y, Toyota J, Ikeda K, Suzuki F, Chayama K, et al. (2013) Characterization of virologic escape in hepatitis C virus genotype 1b patients treated with the direct-acting antivirals daclatasvir and asunaprevir. J Hepatol 58: 646-654.

11. Nettles RE, Gao M, Bifano M, Chung E, Persson A, et al. (2011) Multiple ascending dose study of BMS-790052, a nonstructural protein 5A replication complex inhibitor, in patients infected with hepatitis C virus genotype 1. Hepatology 54: 1956-1965.

12. Eley T, Pasquinelli C, Wendelburg P, Villegas C, He B, et al. (2010) Safety and Pharmacokinetics of BMS-650032 Following Multiple Ascending Doses for 14 Days in Healthy Subjects ICAAC. Boston.

13. Bliss CI (1939) The toxicity of poisons applied jointly. Annals of Applied Biology 26: 585-615.

14. Loewe S, Muischnek H (1926) Combinated effects I Announcement - Implements to the problem. Naunyn-Schmiedebergs Archiv Fur Experimentelle Pathologie Und Pharmakologie 114: 313-326.

15. Ribeiro RM, Bonhoeffer S, Nowak MA (1998) The frequency of resistant mutant virus before antiviral therapy. AIDS 12: 461-465.

16. Fridell RA, Qiu D, Wang C, Valera L, Gao M (2010) Resistance analysis of the hepatitis C virus NS5A inhibitor BMS-790052 in an in vitro replicon system. Antimicrob Agents Chemother 54: 3641-3650.

17. McPhee F, Friborg J, Levine S, Chen C, Falk P, et al. (2012) Resistance analysis of the hepatitis C virus NS3 protease inhibitor asunaprevir. Antimicrob Agents Chemother 56: 3670-3681.

18. Ke R, Lloyd-Smith JO (2012) Evolutionary analysis of human immunodeficiency virus type 1 therapies based on conditionally replicating vectors. PLoS Comput Biol 8: e1002744.
