# Supplementary figures and images for: Rational Design and Adaptive Management of Combination Therapies for Hepatitis C Virus Infection
Source: PLoS Comput Biol. 2015 Jun 30;11(6):e1004040. doi: 10.1371/journal.pcbi.1004040 (PMC4488346; doi:10.1371/journal.pcbi.1004040)

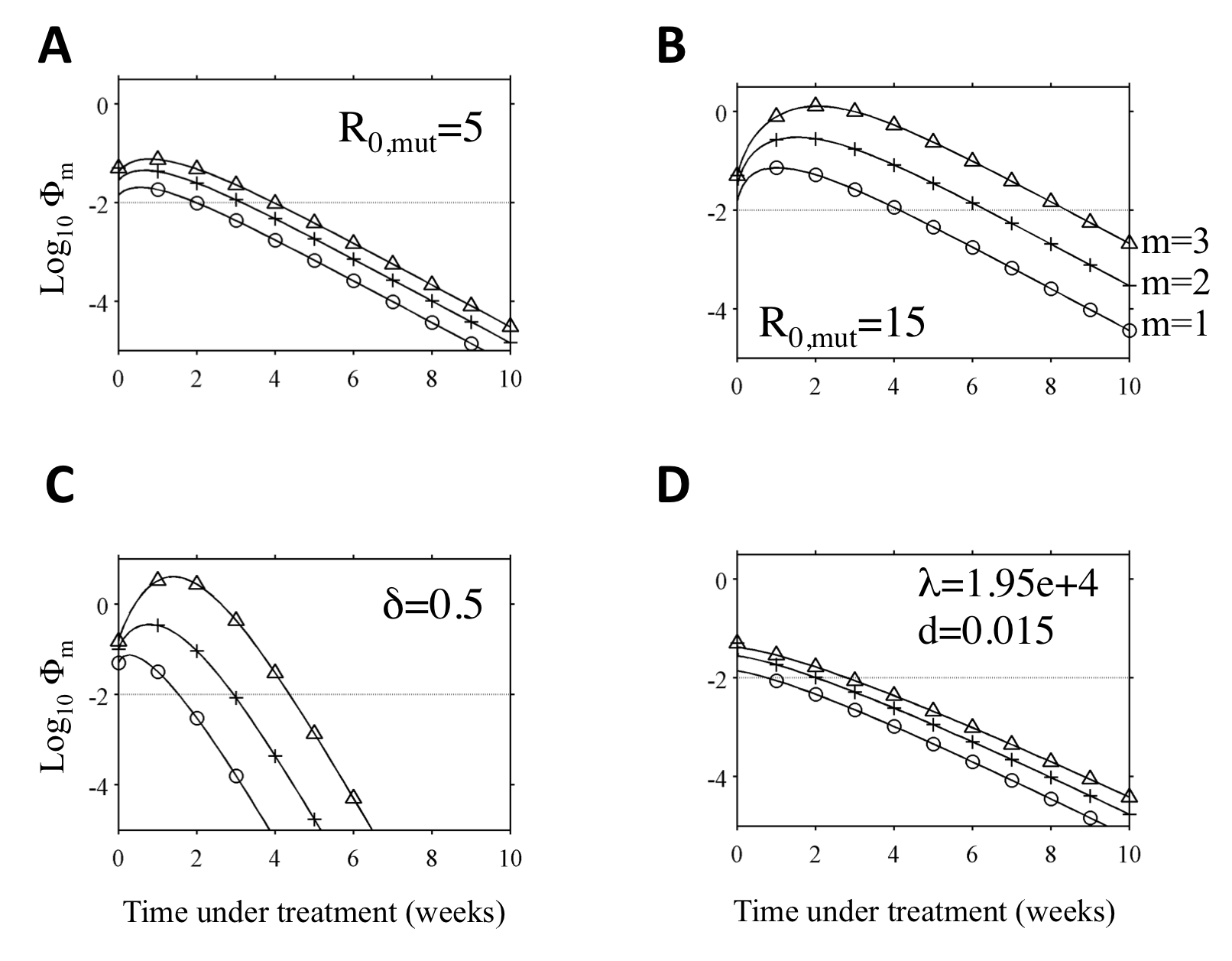

Supplement: S1 Fig — In each panel, the trajectories show how the risk of de novo resistance (Log10Φm) changes over time if adherence is perfect. Figures are plotted using the same parameter settings as trajectories ‘a’ in Fig 3 in the main text, except that R0,mut = 5 in panel A, R0,mut = 15 in panel B, δ = 0.5 in panel C and α = 1.95*104, d = 0.015 in panel D. In the main results, i.e. Fig 3, the parameter values used are R0,mut = 10, δ = 0.15, λ = 1.95*105, d = 0.15. (TIFF) [file pcbi.1004040.s002.tiff]

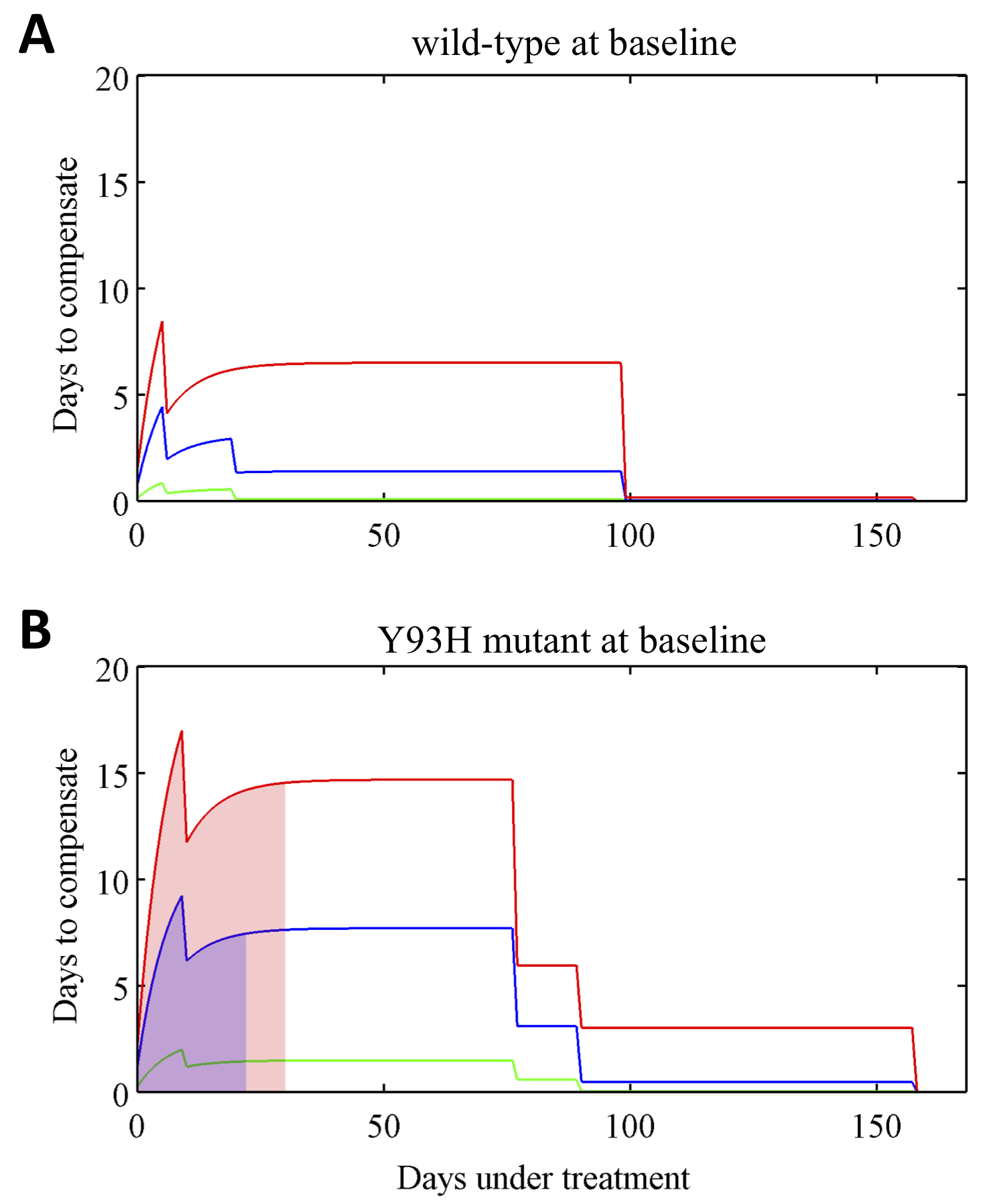

Supplement: S2 Fig — (A) Predictions for patients with the wild-type virus at baseline before treatment. The areas below the curves are white, indicating that the risk of de novo resistance is always low. (B) Predictions for patients with the Y93H mutant virus at baseline. The initial increases of the number of days of compensating doses are due to the increase of the number of target cells upon treatment, and the sudden drops during later periods of treatment are due to the elimination of particular partially resistant mutant lineages. Note that these curves are calculated under the assumption that adherence is perfect except for the 1–3 days of missed doses being considered, i.e. it is a prediction for the first instance of missed doses. For cases where multiple instances of missed doses have occurred, one needs to calculate the values of Nm and Φm for each mutant based on the adherence pattern, and then integrate them together by choosing the highest values of Nm and Φm for those mutants. (TIFF) [file pcbi.1004040.s003.tiff]

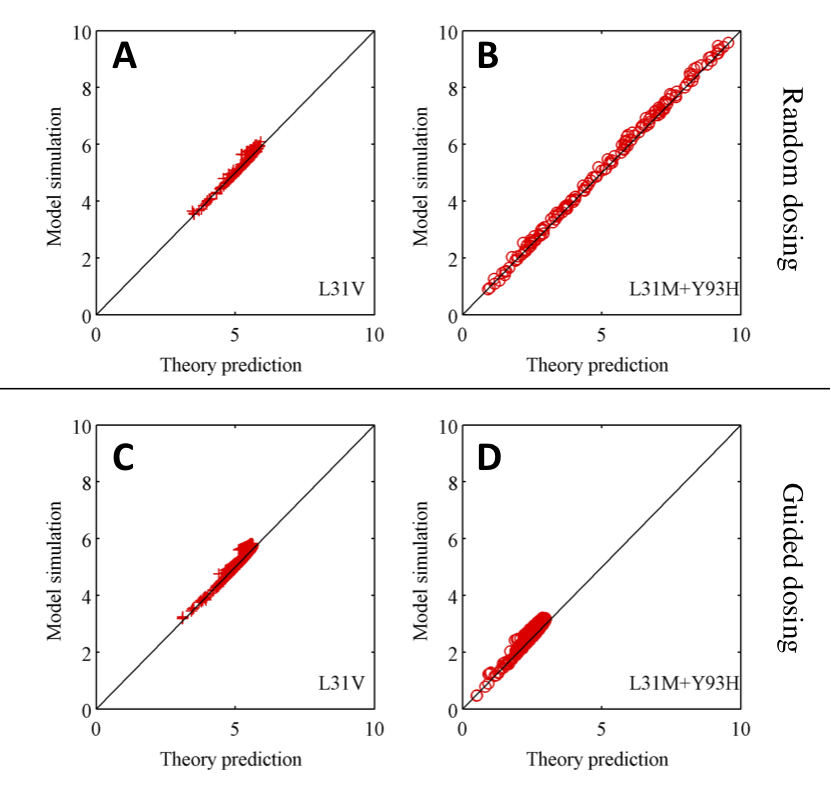

Supplement: S3 Fig — L31V and L31M+Y93H are the two most likely mutants that generate full resistance. The axes are the theory prediction (x-axis) and model simulation (y-axis) of the Log10 of the number of mutants, which are calculated as the cumulative numbers of Log10 Φ m(t)/μ mut for all missed doses. (TIFF) [file pcbi.1004040.s004.tiff]

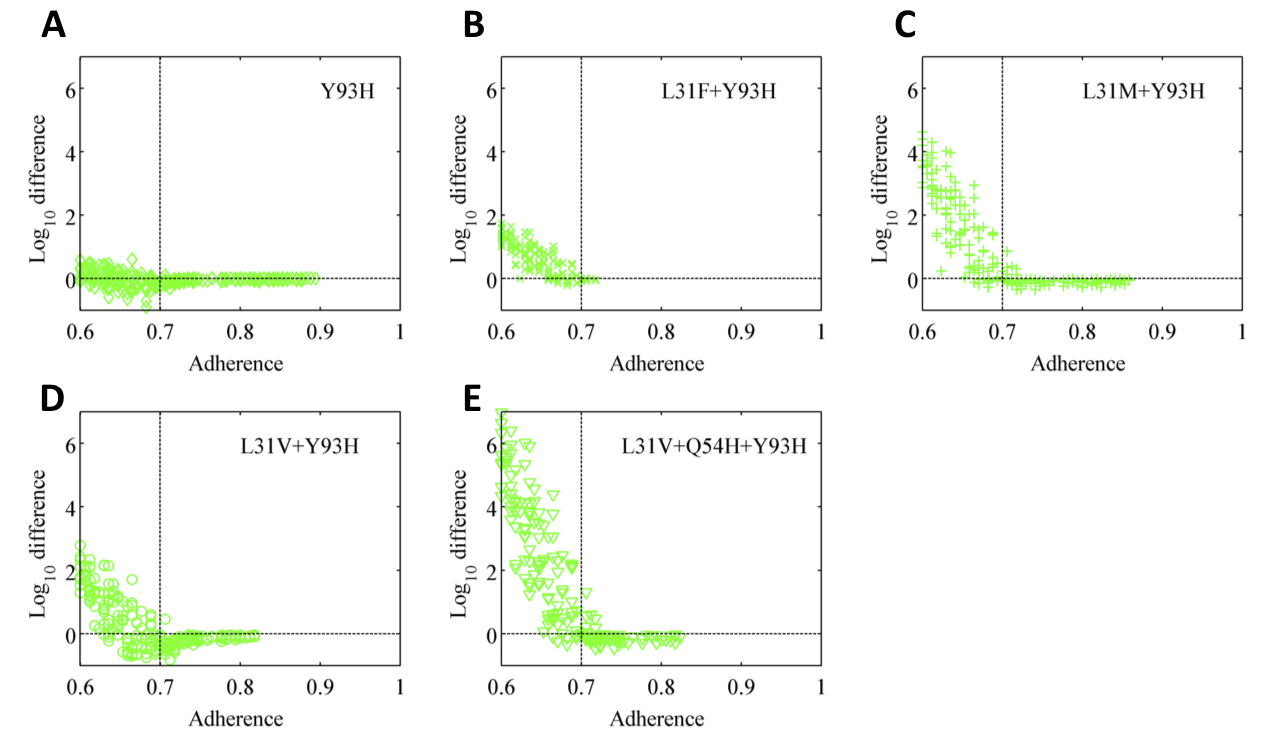

Supplement: S4 Fig — The y-axis shows the log10 difference between the theory prediction and the model simulation at the end of the 24-weeks’ treatment. Note that when adherence is lower than 70%, the population of infected cells grows to high levels close to the pre-treatment level, where further growth is curtailed by target cell limitation. As a result, the theoretical prediction overestimates the number of cells infected by the mutant virus significantly because we assume the number of target cells is not limited. (TIFF) [file pcbi.1004040.s005.tiff]

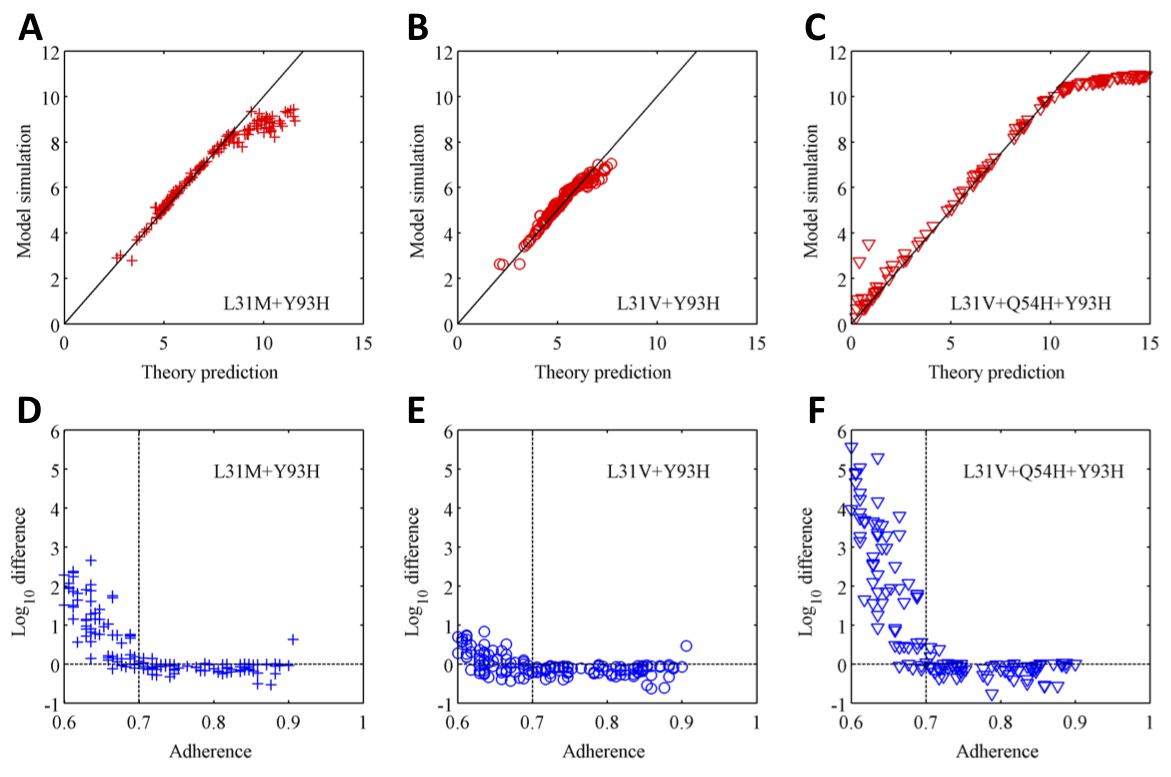

Supplement: S5 Fig — (A,B,C) The axes are the theory prediction (x-axis) and model simulation (y-axis) of the Log10 of the number of cells infected by different mutants, which are calculated as the cumulative numbers of Log10 Φm/μmut for all missed doses (L31M+Y93H in panel A; L31V+Y93H in panel B; L31V+Q54H+Y93H in panel C). (D,E,F) Our theory prediction is accurate for adherence greater than 70%, but overestimates the number of cells infected by the mutant virus significantly when adherence is lower than 70%, for the same reason as explained in the legend of S4 Fig. (TIFF) [file pcbi.1004040.s006.tiff]

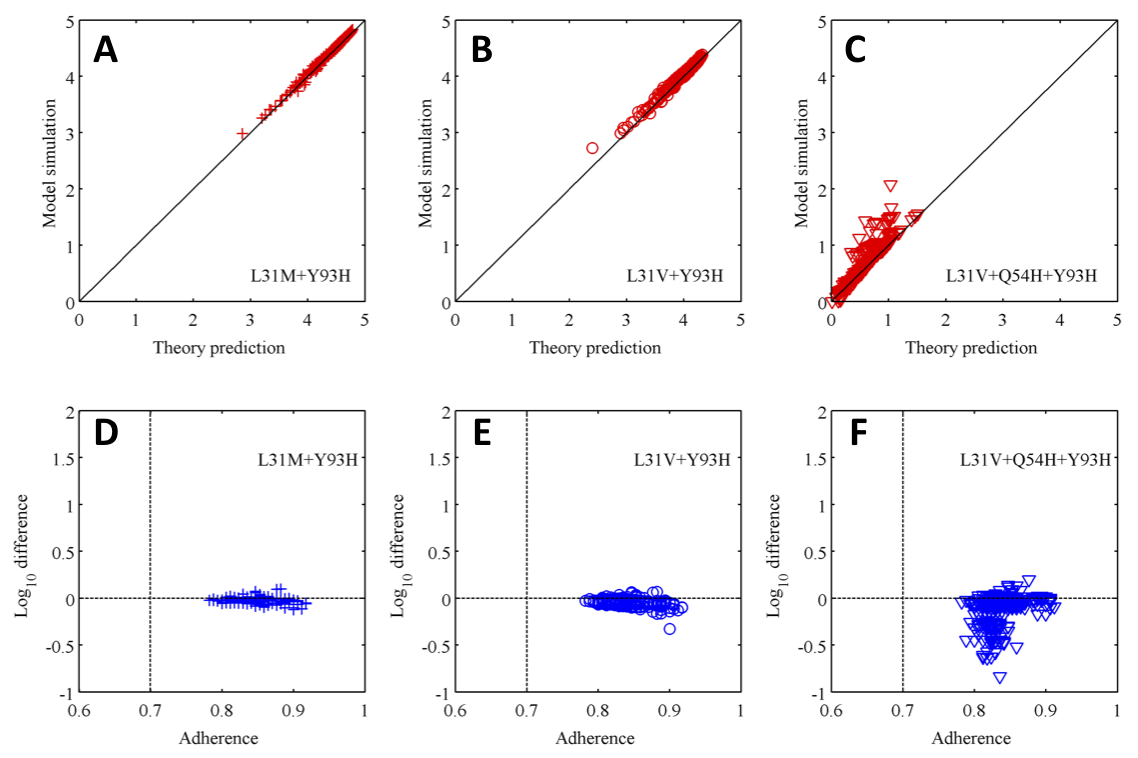

Supplement: S6 Fig — (A,B,C) The axes are the theory prediction (x-axis) and model simulation (y-axis) of the Log10 of the number of mutants (L31M+Y93H in panel A; L31V+Y93H in panel B; L31V+Q54H+Y93H in panel C), which are calculated as the cumulative numbers of Log10 Φm(t)/ μmut for all missed doses. (D,E,F) the Log10 differences between theory prediction and model simulation as shown in panels (A,B,C). Note that our theory agrees very well for mutants L31M+Y93H and L31V+Y93H. For mutant L31V+Q54H+Y93H, the stochastic extinction and appearance of this mutant generates stochastic deviations of the simulation from theory prediction. (TIFF) [file pcbi.1004040.s007.tiff]

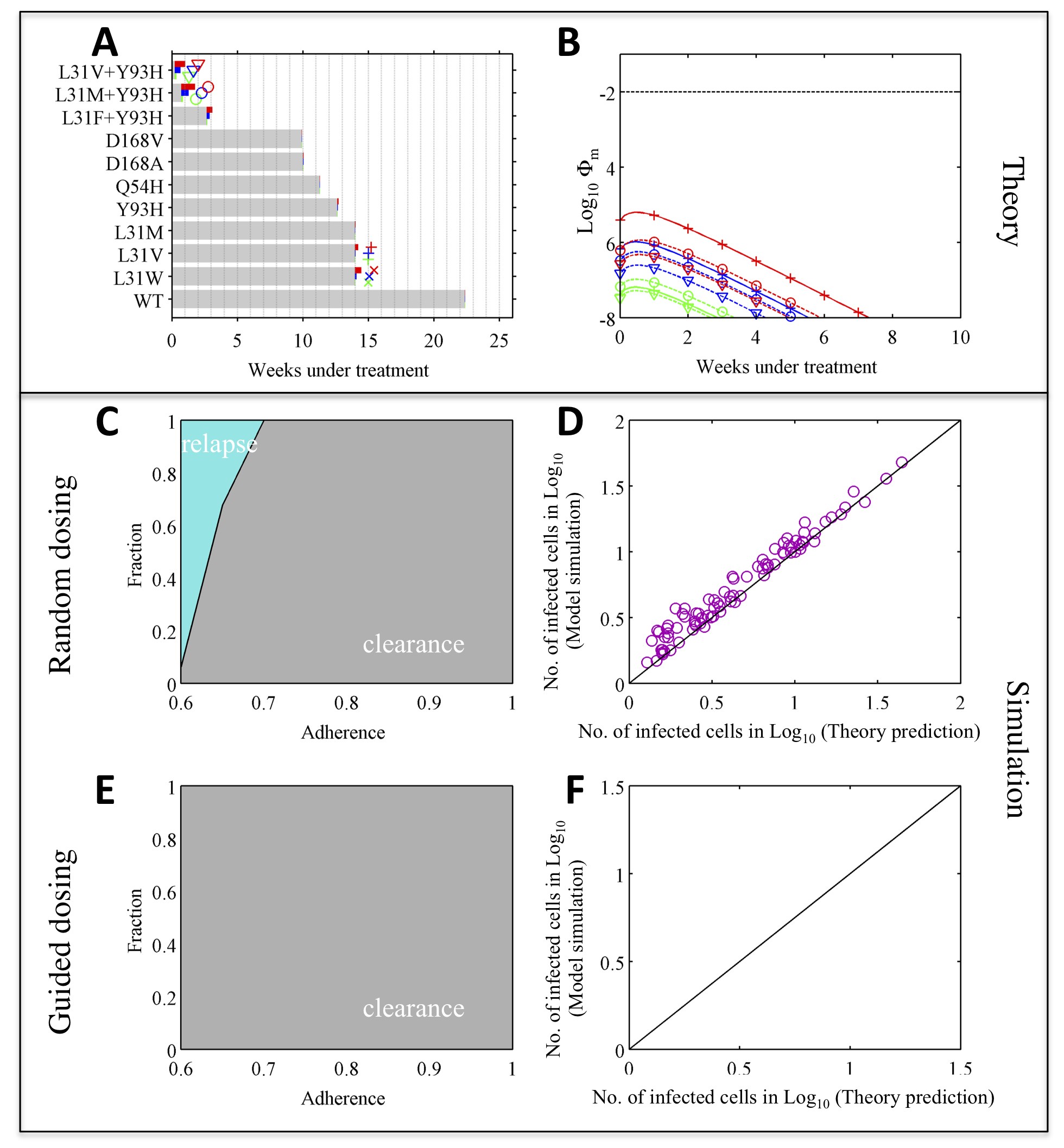

Supplement: S7 Fig — Panels A-F show the same plots as Fig 4 in the main text, except that the fitness parameter R0 for the wild-type virus is assumed to be 5. The treatment outcome improves for all scenarios for this lower viral fitness (compare with Fig 4 in the main text). Using the adaptive treatment strategy prevents viral relapse and de novo resistance if overall adherence is greater than 60% (panel E). Panel F is empty because all patients are cleared of infection after 24 weeks. (TIFF) [file pcbi.1004040.s008.tiff]

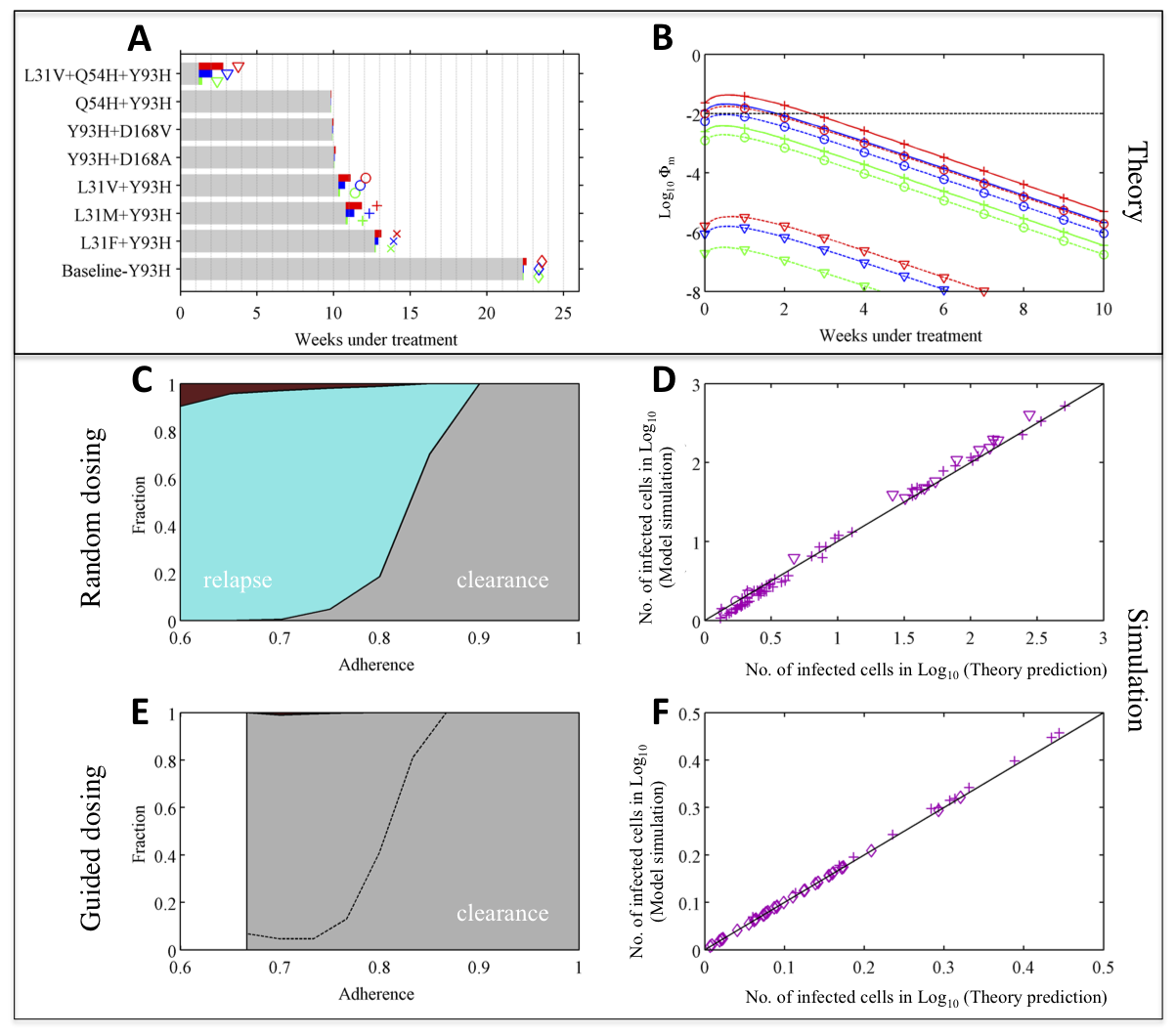

Supplement: S8 Fig — Panels A-F show the same plots as Fig 5 in the main text, except that the fitness parameter R0 for the wild-type virus is assumed to be 5. The treatment outcome improves for all scenarios for this lower viral fitness (compare with Fig 5 in the main text). Using adaptive treatment strategy reduced the risk of de novo resistance (panels E). Our theory correctly predicts the number of infected cells in a patient at the end of 24 weeks’ treatment. (TIFF) [file pcbi.1004040.s009.tiff]

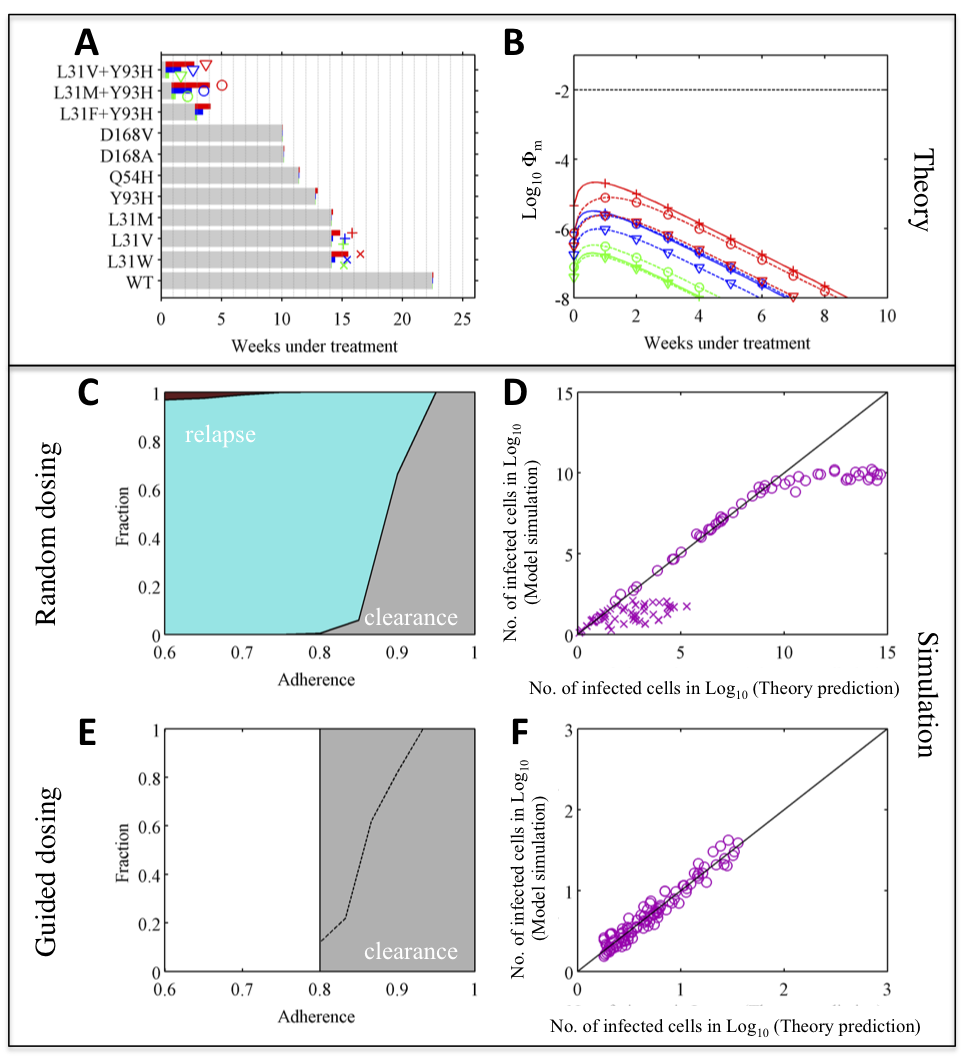

Supplement: S9 Fig — Panels A-F show the same plots as Fig 4 in the main text, except that the fitness parameter R0 for the wild-type virus is assumed to be 15. The treatment outcome improves for all scenarios for this lower viral fitness (compare with Fig 4 in the main text). Our theory correctly predicts the number of infected cells in a patient at the end of 24 weeks’ treatment. (TIFF) [file pcbi.1004040.s010.tiff]

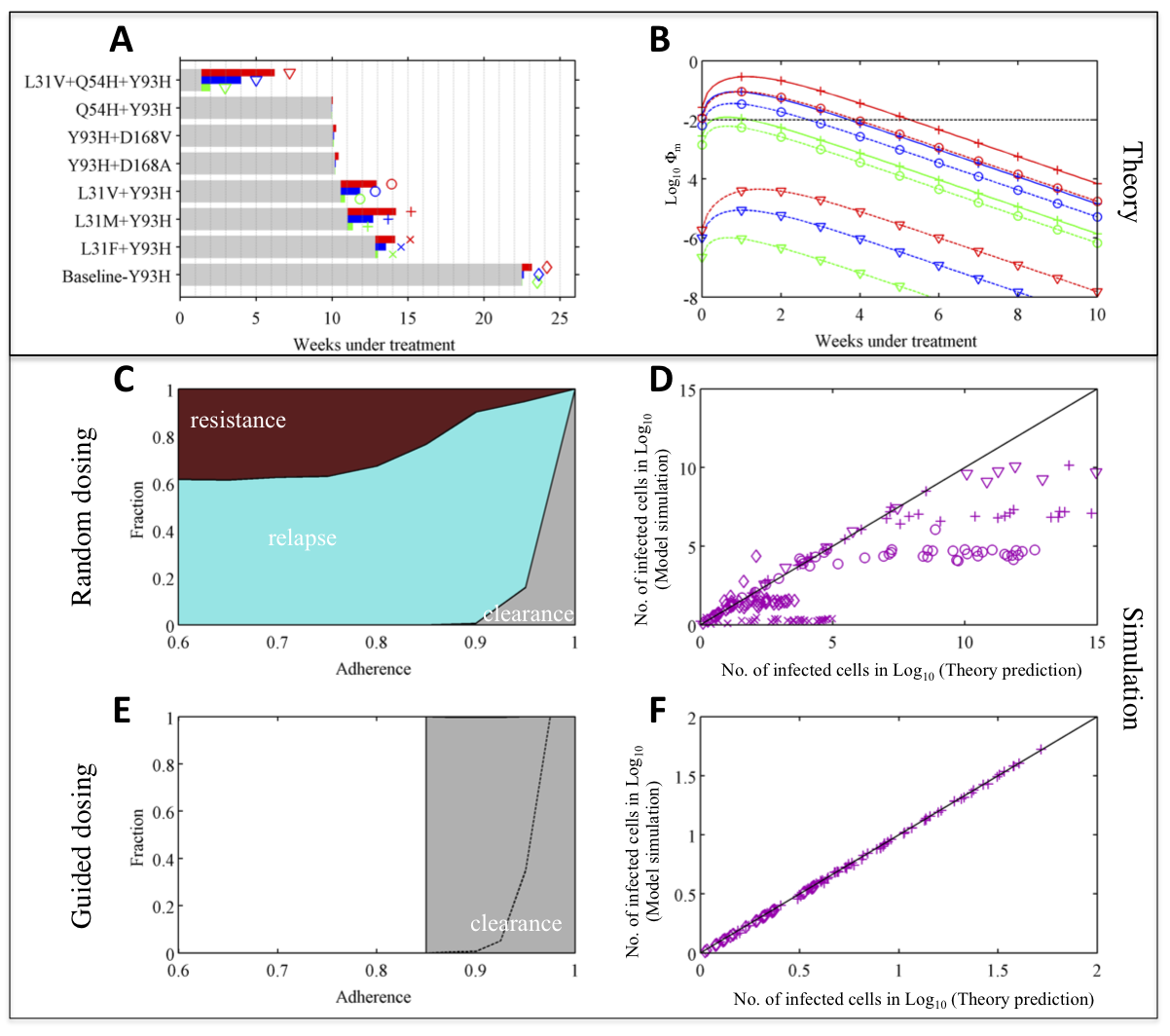

Supplement: S10 Fig — Panels A-F show the same plots with Fig 5 except that in the analytical derivation and model simulation, the fitness parameter R0 for the Y93H mutant virus is assumed to be 15. The risks of viral relapse and de novo resistance become higher when the viral fitness, R0, is higher. Using adaptive treatment strategy can prevent de novo resistance and improve treatment outcomes (panels E and F). Our theory correctly predicts the number of infected cells in a patient at the end of 24 weeks’ treatment when doses are guided (panels F). Our theory does not predict the number of infected cells at the end of treatment well, when doses are missed randomly and the adherence is low. This is because, when adherence is low, the viral load often rebounds back to the pre-treatment level, where it is limited by target cell availability. This phenomenon is not included in our theory, which overestimates the number of viruses as a result. (TIFF) [file pcbi.1004040.s011.tiff]

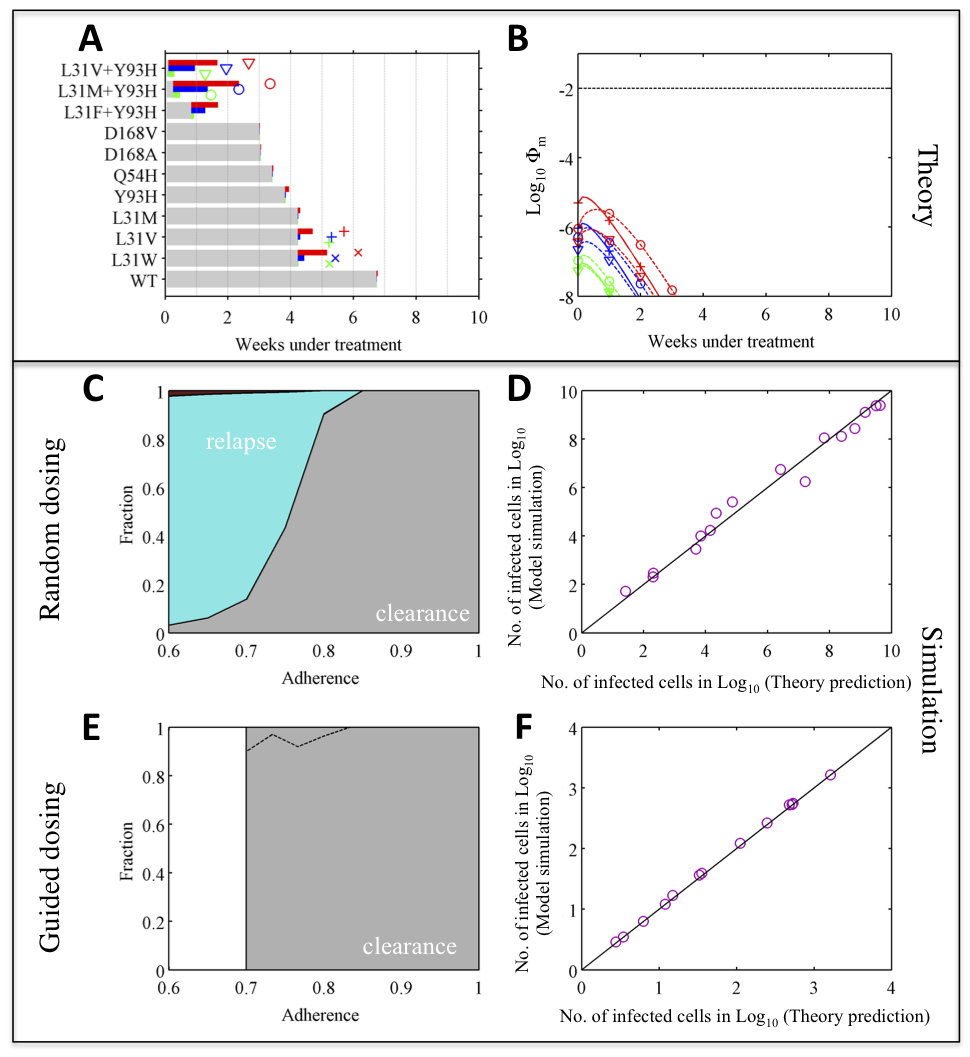

Supplement: S11 Fig — Panels A-F show the same plots with Fig 4 except that in the analytical derivation and model simulation, the viral clearance rate, δ, is assumed to be 0.5 instead of 0.15 in Fig 4 (but note that R0 for the viruses is kept the same). When the viral clearance rate increases, it takes less time to eradicate the virus from a patient. However, when doses are missed, the population of mutant viruses expands more quickly, because the half-life of the infected cells is shorter and thus it undergoes a higher number of replication generations during the period of missed doses. Using the adaptive treatment strategy can prevent viral relapse and de novo resistance and improve treatment outcome (panels E). Our theory correctly predicts the number of infected cells in a patient at the end of 24 weeks’ treatment when doses are guided (panel D). (TIFF) [file pcbi.1004040.s012.tiff]

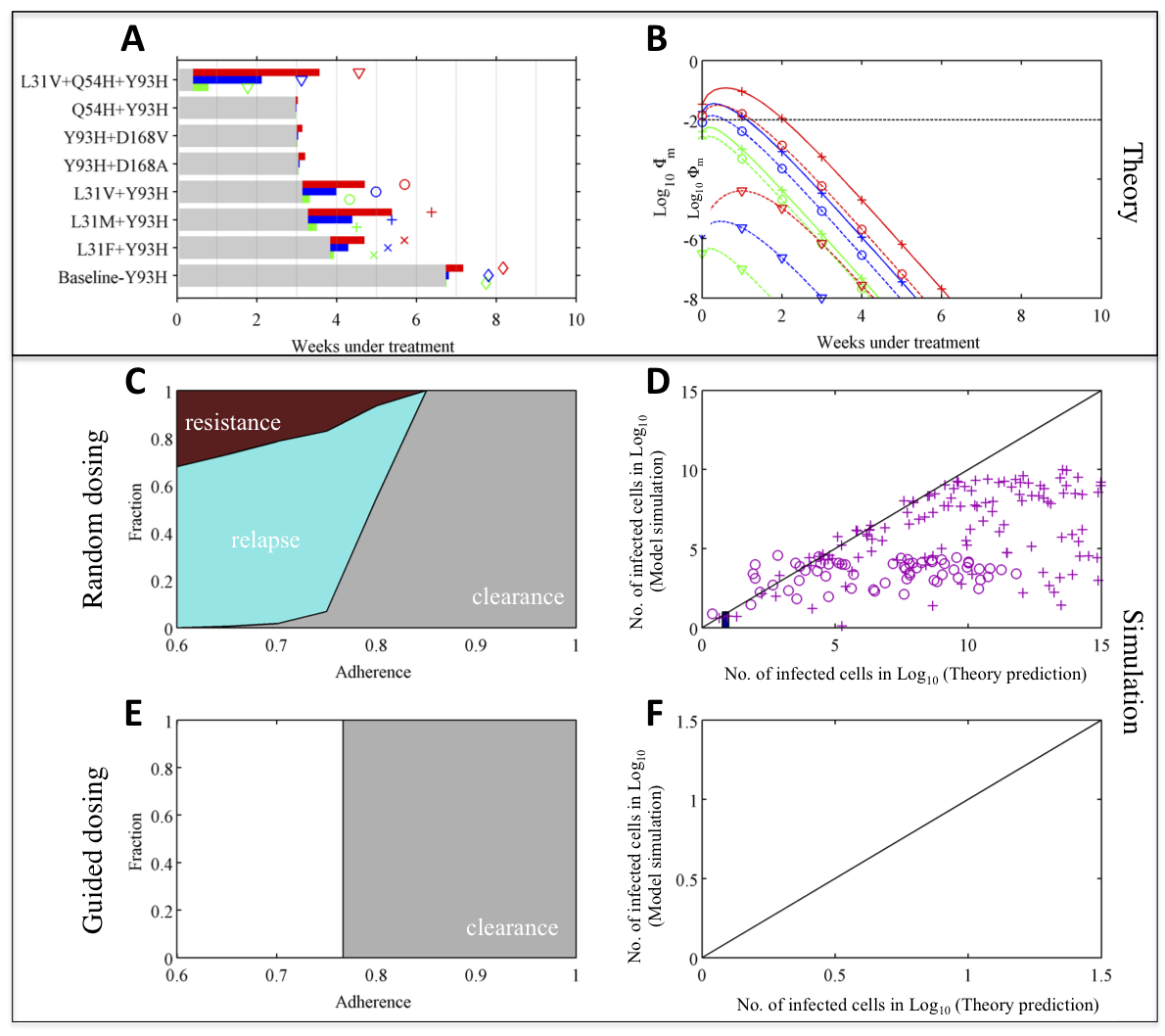

Supplement: S12 Fig — Panels A-F show the same plots with Fig 5 except that in the analytical derivation and model simulation, we assume the viral clearance rate, δ, is 0.5 instead of 0.15 (but note that R0 for the viruses is kept the same). As seen in S11 Fig for the scenario with the wild-type virus at baseline, it takes less time to eradicate the virus from a patient for this higher viral clearance rate. However, when doses are missed, the population of mutant viruses expands more quickly, increasing the risk of viral relapse and de novo resistance. Using adaptive treatment strategy can prevent viral relapse, de novo resistance and improve treatment outcome (panels E and F). Our theory does not predict the number of infected cells at the end of treatment well, when doses are missed randomly and adherence is low. This is because, during the time period when doses are missed, the rebound of the viruses is quicker when δ is higher (because the viral generation time is shorter). When adherence is low, the viral load often rebounds back to the pre-treatment level, where it is limited by target cell availability. This phenomenon is not included in our theory, which overestimates the number of viruses as a result. (TIFF) [file pcbi.1004040.s013.tiff]
